# Fyn depletion ameliorates tau^P301L^-induced neuropathology

**DOI:** 10.1186/s40478-020-00979-6

**Published:** 2020-07-14

**Authors:** Guanghao Liu, Kimberly L. Fiock, Yona Levites, Todd E. Golde, Marco M. Hefti, Gloria Lee

**Affiliations:** 1 Interdisciplinary Program in Neuroscience, University of Iowa Carver College of Medicine, Iowa City, IA USA; 2 Department of Pathology, University of Iowa Carver College of Medicine, Iowa City, IA USA; 3 Department of Neuroscience, Center for Translational Research in Neurodegenerative Disease, University of Florida, Gainesville, FL USA; 4 Department of Internal Medicine, University of Iowa Carver College of Medicine, 500 Newton Road, ML B191, Iowa City, IA 52242 USA

**Keywords:** Fyn, Tau, Neurofibrillary tangles

## Abstract

The Src family non-receptor tyrosine kinase Fyn has been implicated in neurodegeneration of Alzheimer’s disease through interaction with amyloid β (Aβ). However, the role of Fyn in the pathogenesis of primary tauopathies such as FTDP-17, where Aβ plaques are absent, is poorly understood. In the current study, we used AAV2/8 vectors to deliver tau^P301L^ to the brains of WT and Fyn KO mice, generating somatic transgenic tauopathy models with the presence or absence of Fyn. Although both genotypes developed tau pathology, Fyn KO developed fewer neurofibrillary tangles on Bielschowsky and Thioflavin S stained sections and showed lower levels of phosphorylated tau. In addition, tau^P301L^-induced behavior abnormalities and depletion of synaptic proteins were not observed in the Fyn KO model. Our work provides evidence for Fyn being a critical protein in the disease pathogenesis of FTDP-17.

## Background

Alzheimer’s disease (AD), along with a subset of disorders such as frontotemporal dementia with Parkinsonism linked to chromosome 17 (FTPD-17), corticobasal degeneration (CBD), progressive supranuclear palsy (PSP), and Pick’s disease, exhibit neurofibrillary tangles (NFTs) made up of aggregated hyperphosphorylated tau and are collectively known as tauopathies (reviewed by [78]). During the disease process, tau becomes abnormally hyperphosphorylated, detaches from microtubules, undergoes conformational changes, and ultimately aggregates to form NFTs (reviewed by [1, 21, 50, 52, 73]). AD also contains an additional neuropathological hallmark, amyloid β (Aβ) plaques. Although no mutations in the tau gene have been identified to cause AD, several mutations in tau, such as P301L, have been found to cause FTDP-17, where tau hyperphosphorylation, neurofibrillary tangle formation, and neurodegeneration occur in the absence of Aβ plaques [12, 29, 60, 79].

In neurons, tau is enriched in axons to regulate microtubule assembly (reviewed by [17, 36, 81]). We have previously reported that tau can also associate with the cytoplasmic face of the plasma membrane [7] and interacts with the SH3 domain of Src family non-receptor tyrosine kinases (SFK) such as Fyn and Src [41]. We found that Fyn phosphorylates tau at tyr18 [42] and that the tau-Fyn interaction increased the auto-phosphorylation of Fyn as well as the enzymatic activity of Fyn [71]. In addition, we reported that tau^P301L^ and other FTDP-17 tau mutants had a higher binding affinity to the Fyn SH3 domain, when compared to WT tau [4]. Subsequently, it was discovered that tau is also involved in targeting Fyn to the postsynaptic space in dendrites; Fyn then affects synaptic plasticity by phosphorylating the *N*-methyl-d-aspartate receptor (NMDAR) which facilitates the interaction between NMDAR and postsynaptic density 95 protein (PSD-95) [32, 56, 63]. It has also been reported that tau^P301L^ is selectively enriched in dendritic spines whereas WT tau is not [27, 82].

Initially, Fyn was linked to AD when it was discovered that neuritic plaques and dystrophic neurites in AD brain contained phospho-tyrosine and that Fyn was up-regulated in a subset of neurons and co-localized with NFTs in AD brain [26, 72, 90]. Subsequent studies using cell culture showed that Aβ treatment increased both Fyn activation and the level of tyrosine phosphorylated tau [48, 89]. In addition, genetic ablation of Fyn was protective against oligomeric Aβ-mediated neurotoxicity [39] while overexpressing Fyn in human amyloid precursor protein transgenic mice increased synaptic and cognitive impairments [10, 11]. Furthermore, inhibiting Fyn activity through a small molecule inhibitor in the triple transgenic AD mouse model also significantly rescued memory and cognitive impairments [33]. The mechanism by which Aβ activates Fyn involves the binding of soluble Aβ oligomers to cellular prion protein (PrPC) [85, 86]. The Aβ-PrPC complex then interacts with the metabotropic glutamate receptor (mGluR5) to activate intracellular Fyn kinase [84]. The effect of Aβ on Fyn activation has been linked to synaptic abnormality and behavioral deficits in APP mouse models (reviewed by [5, 6, 75]. Fyn has also been directly and indirectly implicated in the hyperphosphorylation of tau at disease-related sites. Firstly, Fyn has been reported to activate Cdk5 [67] and GSK-3 [43], two kinases that hyperphosphorylate tau on serine and threonine residues [24, 25, 47, 49, 58]. Secondly, we have found that phospho-tyr18-tau is present in neurofibrillary tangles in brains from AD patients [42] as well as brains from an FTDP-17 mouse model that expresses the tau^P301L^ mutation [2, 4]. In addition, pY18-tau also co-localized with activated Src family tyrosine kinases (SFK) in NFTs of FTDP-17 mouse brains [2]. In Aβ-mediated neurodegeneration, the importance of the tau-Fyn interaction has been supported by the finding that inhibition of the interaction ameliorated the toxic effects of Aβ oligomers [65].

In FTDP-17 mouse models, several changes induced by tau^P301L^ have been reported, such as behavioral deficits, tau hyperphosphorylation, electrophysiological changes, and structural atrophy of dendritic spines with reduction of surface NMDA and AMPA receptors [16, 27, 28, 37, 59, 62, 64, 66]. However, the role played by Fyn in the disease pathogenesis of FTDP-17, where Aβ is absent, is unknown. In this study, we have used a viral gene transduction method to facilitate somatic brain transgenesis and to create an FTDP-17 tauopathy model on a Fyn KO background. We found that depleting Fyn reduced tau-related pathology. In the absence of Fyn, tau^P301L^-induced neurofibrillary tangle burden and tau hyperphosphorylation at disease related sites were decreased. Fyn depletion also reduced the neuroinflammatory cell activation caused by the mutant tau. Also, tau^P301L^-induced behavior abnormalities and depletion of synaptic proteins were not observed in the Fyn KO tauopathy model. Our results establish Fyn as a critical protein involved in the pathogenesis of FTDP-17.

## Methods

### Mice

Fyn KO (C57BL/6;S129) mice were obtained from Jackson Laboratories (strain 002385). WT mice were generated by crossing the Fyn KO and Tau KO (C57BL/6, strain 007251) mice, as described [46]. In this way, the strain of the WT would be closer to that of the Fyn KO, minimizing strain related differences. Litter mate controls were not used. All procedures and animal care were approved by the University of Iowa Institutional Animal Care and Use Committee and in compliance with the *NIH Guide for the Care and Use of Laboratory Mice.*

### AAV-tau^P301L^ injection at postnatal day 0 (p0)

AAV2/8-tau^P301L^, produced by the University of Iowa Viral Vector Core, was injected intracerebroventricularly (ICV) into WT and Fyn KO mouse pups on postnatal day 0 (4.2e12 viral particles/μl; 2 μl/ventricle). ICV injections were performed as described [9], where newborn mice were anesthetized by placing on a cold pack and a 32-gauge needle was used to pierce the skull 2/5 way between eyes and bregma and inserted at 0.5 cm depth. Both right and left lateral ventricles were each injected with 2 μl of AAV. At 6 months of age, behavioral testing was performed. Subsequently, mice were deeply anesthetized with isofluorane then perfused with phosphate-buffered saline. The brain was removed and bisected along the midline. Half was drop-fixed in 4% paraformaldehyde overnight at 4 °C for histology, whereas the other half was frozen in liquid N_2_ after discarding the olfactory bulb, cerebellum, and midbrain. WT mice and Fyn KO mice injected with AAV-tau^P301L^ were designated WT-AAV and Fyn KO-AAV, respectively. In AAV2/8, the expression of tau^P301L^ was regulated by a cytomegalovirus enhancer/chicken beta actin (CBA) promoter, a woodchuck hepatitis virus post-transcriptional-regulatory element (WPRE) and the bovine growth hormone polyA [9].

### Magnetic resonance imaging

Mice were imaged at 8 weeks of age. Varian Unity/Inova 4.7 T small-bore MRI system (Varian, Inc., Palo Alto, CA; Small Animal Imaging Facility, University of Iowa) with an in-plane resolution of 0.13 × 0.25 mm^2^ and 0.6 mm slice thickness was used. Coronal images were collected and lateral ventricle volume sizes were analyzed with ImageJ [8]. Quantification was carried out as previously described [46]. Fyn KO mice were divided by degree of hydrocephalus, with lateral ventricle volume of ≤0.2 units considered normal, 0.2 < x ≤ 2 units as moderate, and > 2 units as severe hydrocephalus. The lateral ventricle volume (LLV) cut-offs used to define these groups were based on the total movement assay, where significant differences in movement occurred between the groups as defined by these LLV cut-offs.

### Preparation of brain homogenates, crude synaptosomes, and western blot analysis

Mouse hemi-brains were homogenized in sucrose buffer (0.32 M sucrose, 1 mM NaHCO_3_, 1 mM MgCl_2_, 0.5 mM CaCl_2_, 10 mM NaF, 1 mM NaVO_4_, 1 mM AEBSF, 10 μg/ml pepstatin, 10 μg/ml leupeptin, 10 μg/ml aprotinin) and samples were centrifuged (1000 g, 10 min, 4 °C). The pellet, containing nucleus and cell debris, was discarded and the supernatant, containing cytosolic proteins, was retained as the crude brain lysate. An equal amount of 2X Laemmli sample buffer was added and heat-denatured for 5 min at 95 °C.

Western blotting of crude brain lysates has been previously described [41]. Primary antibodies used were: GAPDH (1: 20,000 mouse monoclonal, Chemicon); Fyn3 (1: 1000 rabbit polyclonal, Santa Cruz); tau5-HRP (1: 10,000, [44]); tau13 (1: 20,000 mouse monoclonal, gift from late Dr. Lester Binder); AT8 (1: 1000 mouse monoclonal, ThermoFisher); anti-pY18-tau (1:1000 rabbit polyclonal [42]); PSD95 (1:1000 rabbit polyclonal, Millipore Sigma); and NeuN (1:5000 mouse monoclonal, Millipore Sigma). Quantification of lightly exposed blots was done by densitometry using ImageJ. For Fig. 3, total tau levels from crude lysates, measured by tau5, were normalized to GAPDH levels; AT8 and pY18 signals were normalized to tau5 and protein level values from Fyn KO-AAV were normalized to those from WT-AAV. 19 WT-AAV and 7 Fyn KO-AAV mice were used for AT8 and tau5; 12 WT-AAV and 7 Fyn KO-AAV mice were used for anti-pY18-tau. For quantitating NeuN and PSD95 (Fig. 6), levels were first normalized to GAPDH and then normalized to WT-uninjected mice. At least 7 WT, 9 WT-AAV, 9 Fyn KO, and 9 Fyn KO-AAV mice were used.

Crude synaptosome preparations were prepared from the crude brain lysate as previously described [32]. Primary antibodies used to probe the crude synaptosome preparations were: Fyn3, tau5-HRP, and tau13 (as above); β-actin (JLA20 1:10,000 mouse monoclonal, Iowa DSHB); PSD-95 (EP2652Y, 1:1000 rabbit monoclonal, Millipore, Cat# 04–1066); and NR2B (1:1000 mouse monoclonal, One World Labs, StressMarq, Cat # SMC-33D). Quantification of lightly exposed blots was done by densitometry using ImageJ. PSD95, NR2B, and Fyn levels were normalized to β-actin levels. Protein level values from WT-AAV, Fyn KO, and Fyn KO-AAV were normalized to those from WT with the WT values represented as “1”. 7 WT, 10 WT-AAV, 6 Fyn KO, and 11 Fyn KO-AAV mice were used for synaptosome preparations.

### Histology and immunohistochemistry

Hemi-brains fixed in 4% paraformaldehyde were embedded in paraffin, sectioned using a sagittal plane at 5-μm thickness, and mounted on glass slides. The tissue sections were deparaffinized in xylene and rehydrated in a graded series of alcohols. Antigen retrieval was performed by steaming in 1 M Tris pH 9 buffer for 30 min. Sections were stained using Bielschowsky silver stain or tau13 (1:5000 mouse monoclonal, gift from late Dr. Lester Binder). Sections was also labeled with 0.05% Thioflavin S. Sections were examined with light or epifluorescence microscopy (Olympus BX61). 3 WT-AAV and 3 Fyn KO-AAV mice were examined. For quantitating Bielschowsky tangles, 3 random 400x fields containing the highest total tangle burden were used for each mouse and the total number of tangles was counted. 3 WT-AAV and 3 Fyn KO-AAV mice were examined.

9G3 (pY18, 1:1000 ms IgG_2a_) and AT8 (pS199/S202, 1:1000 ms IgG_1_) or CP17 (pT231, 1:1000 ms IgG_3_, gift from Dr. Peter Davies) were used for double labeling immunofluorescence experiments using sections from the same animals. Secondary antibodies used were Rhodamine goat anti-mouse IgG_2a_ (1: 250, SouthernBiotech) for 9G3, FITC goat anti-mouse IgG_1_ (1:250, SouthernBiotech) for AT8, and Alexa 488 goat anti-mouse IgG (1:250, Molecular Probes). Sections were analyzed by epifluorescence microscopy (Nikon E800); to quantitate intensity, integrated optical density (IOD) was obtained from equally exposed images using ImageJ. Four random pictures taken from the hippocampus of 3 WT-AAV and 3 Fyn KO-AAV mice were examined.

Microglia or astrocytes were labeled with antibodies against Iba1(1:300 goat polyclonal, Abcam) or GFAP (1:400 mouse monoclonal, MilliporeSigma), respectively. The sections were also co-labeled with tau13 (1:5000 mouse monoclonal, gift from late Dr. Lester Binder) to show the expression of human tau^P301L^. For analyzing microglia, the entire hippocampus was imaged and the total number of Iba1 positive cells was manually counted. For analyzing astrocytes, the entire hippocampus was imaged and the GFAP positive area was thresholded, then converted to a percent positive area as described previously [74]. Three animals from each of the four groups were analyzed. To determine the number of neurons, sections were labeled with anti-NeuN (1:5000 mouse monoclonal, Millipore Sigma), and based on both NeuN positivity and morphology, neurons were counted from one 400X field in Ammon’s Horn for each mouse. 3 WT, 3 WT-AAV, 3 Fyn KO, and 4 Fyn KO-AAV mice were examined.

### Open field activity

The open field test was performed on 6 month old mice as described previously [15]. Briefly, mice were placed in a 40.6 × 40.6 × 36.8 cm open-field chamber (San Diego Instruments, San Diego, CA), 55 lx, for 20 min. Total activity was defined as total beam breaks throughout the entire box, and central activity was defined as beam breaks occurring in the center (15.2 × 15.2 cm). 8 WT, 21 WT-AAV, 10 Fyn KO, 8 Fyn KO-AAV (mod hydro) mice were used.

### Elevated plus maze

Elevated plus maze was performed on 6 month old mice as described previously [87]. Briefly, a maze was constructed from stainless steel with a Plexiglas base (36 in. tall) and two pairs of arms (2 × 11^5^/_8_ inches) intersecting at right angles. One pair of arms was closed and had six-inch walls on three sides. The two open arms lacked walls. A 2 × 2-in. intersection at the center of the maze connected the four arms. Naive mice were placed onto the center and allowed 5 min to roam freely. The time each animal spent in open arms was recorded. 15 WT, 18 WT-AAV, 10 Fyn KO, and 8 Fyn KO-AAV (mod hydro) mice were used.

### Contextual fear conditioning

Contextual fear conditioning was performed on 6 month old mice as described previously [76], using a near-infrared video-equipped fear conditioning chamber (Med Associates, Inc., St. Albums, VT). On training day (day 1), acquisition of contextual fear conditioning was performed, where a mouse explored the chamber for 3 min and then received 5 shocks (1 s, 0.75 mA), administered 1 min apart through the grid flooring. Total training time was 8 min. On the testing day (day 2), context-evoked freezing was tested by returning the mouse to the conditioning chamber for 6 min without foot shocks. Freezing was defined as an absence of movement other than respiration and scored with VideoFreeze software (Med Associates, Inc.). 12 WT, 20 WT-AAV, 8 Fyn KO, and 9 Fyn KO-AAV (mod hydro) mice were used in Fig. 5. 8 Fyn KO (no hydro), 8 Fyn KO (mod hydro), 9 Fyn KO-AAV (mod hydro) and 6 Fyn KO-AAV (severe hydro) were used in SI Fig. 1B.

### Statistical analysis

Statistical analysis was carried out with GraphPad Prism 7.0. Nonparametric t-test, unpaired parametric t-test, and two-way ANOVA with Tukey’s post hoc multiple comparisons were used to analyze data when appropriate. Mean ± standard error of the mean was used in all figures. *p* ≤ 0.05 was considered as statistically significant, with *p* ≤ 0.05 denoted as *, *p* ≤ 0.01 as **, *p* ≤ 0.001 as ***, and *p* < 0.0001 as ****.

## Results

### Injecting AAV- tau^P301L^ at p0 caused widespread human tau expression

We used intracerebroventricular injection of AAV-tau^P301L^ in P0 mouse pups to create a P301L tauopathy model [14]. As described previously [22], depleting Fyn can predispose mice to develop non-obstructive hydrocephalus. Thus all AAV injected Fyn KO mice were screened with MRI as described in Methods, classifying animals as normal, or with moderate or severe hydrocephalus (examples of MRI images of mice with different levels of hydrocephalus were shown in SI Fig. 1A). All AAV injected Fyn KO mice were found to have some degree of hydrocephalus; of the 22 injected Fyn KO mice, 9 had moderate hydrocephalus and 13 had severe hydrocephalus. In contrast, when 22 AAV injected WT mice were examined, none developed hydrocephalus, indicating that injection of AAV-tau^P301L^ was not sufficient to induce hydrocephalus.

In order to control for the effects of hydrocephalus, Fyn KO-AAV mice with severe hydrocephalus, Fyn KO-AAV with moderate hydrocephalus, and uninjected Fyn KO mice, with moderate hydrocephalus or no hydrocephalus, were compared using behavioral tests. We found that Fyn KO mice with moderate hydrocephalus, either uninjected or injected with AAV, performed similarly to Fyn KO mice without hydrocephalus; only Fyn KO-AAV with severe hydrocephalus had abnormal behavior (SI Fig. 1B). We had also found that the Fyn KO-AAV with severe hydrocephalus had significantly higher levels of hyperphosphorylated tau than both WT-AAV and Fyn KO-AAV with moderate hydrocephalus (SI Fig. 2). Since our goal was to investigate the effects of Fyn KO, rather than the effects of hydrocephalus, Fyn KO-AAV mice with severe hydrocephalus were omitted from the study. Since Fyn KO-AAV with moderate hydrocephalus were similar in behavior to Fyn KO without hydrocephalus (SI Fig. 1B, C), such mice hereafter will simply be referred to as “Fyn KO-AAV”.

Injected mice were harvested at 6 months and the distribution of human tau expression was evaluated histologically. Using immunohistochemistry, human-specific tau13 antibody was used to label human tau (SI Fig. 3). High levels of human tau were detected in the hippocampal regions of both WT-AAV (SI Fig. 3A, D) and Fyn KO-AAV (SI Fig. 3B, E) mice. As expected, uninjected WT mouse brain showed no tau13 labeling (SI Fig. 3C, F). In the previous study using intracerebroventricular injection of AAV-tau^P301L^ in P0 mouse pups, an AAV-GFP was injected as a control, showing that AAV injection alone was not sufficient to cause any of the tau^P301L^-induced effects [14].

### Fyn depletion reduced the effects of tau^P301L^ on NFT formation and abnormal tau hyperphosphorylation

**Fig. 1:**
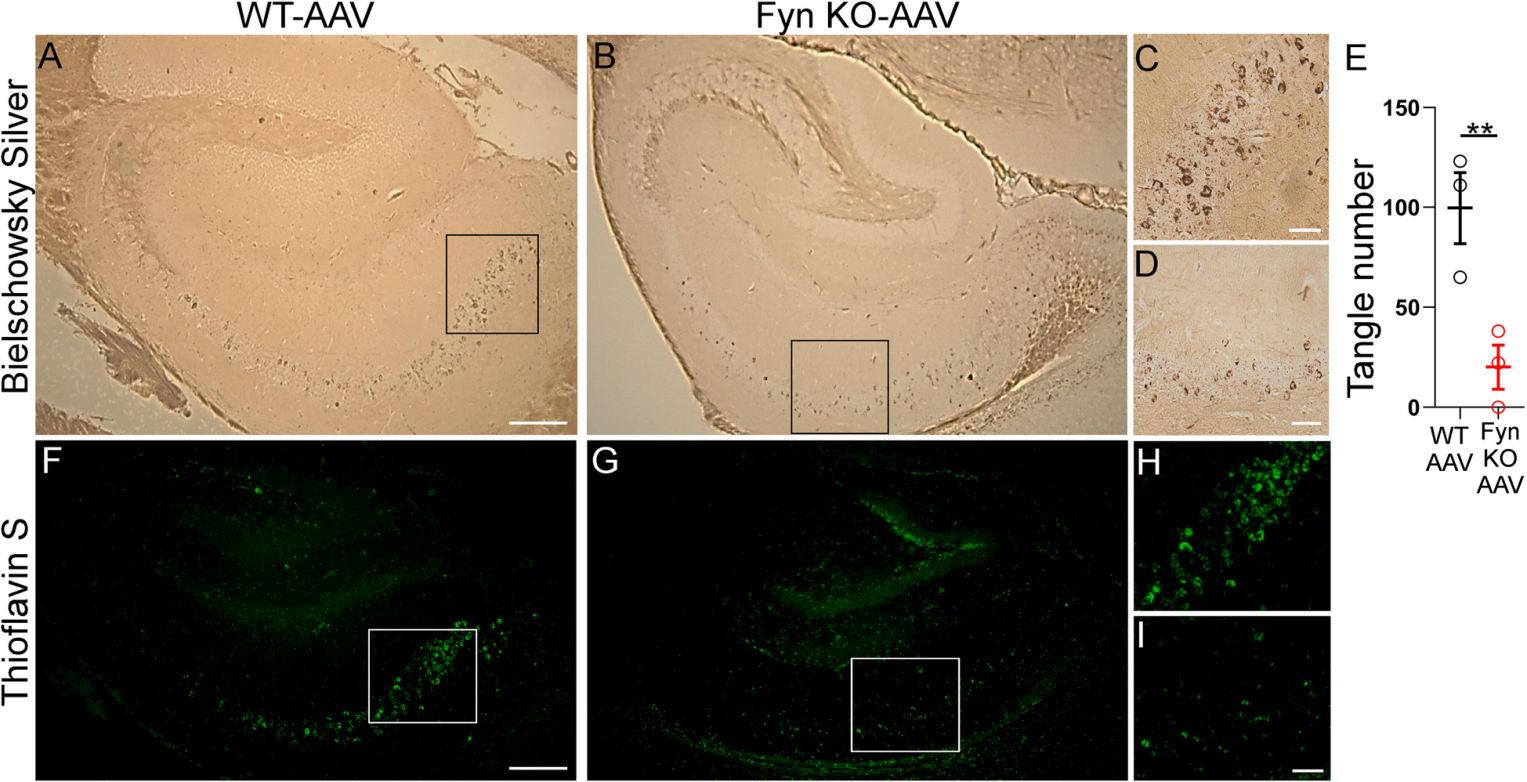
Fyn KO-AAV mice displayed less Bielschowsky silver stained and Thioflavin-S positive NFTs than WT-AAV mice. **a, b, c, d** Hippocampus of WT mice **(a, c)** or Fyn KO mice **(b, d)**, injected with AAV2/8-tau^P301L^, were subjected to Bielschowsky silver staining 6 months post injection. Fyn KO-AAV showed less NFTs than WT-AVV. **a, b** Scale bar: 200 μm. **c** and **d** showed enlarged portions from panels **a** and **b,** respectively; scale bar: 50 μm. **e** Bielschowsky stained tangles were quantitated as described in Methods; three fields from each of six animals was examined. **p = 0.0187, as calculated by t-test. **f, g, h, i** Hippocampus of WT mice (**f**, **h**) or Fyn KO mice (**g**, **i**), injected with AAV2/8-tau^P301L^, were subjected to Thioflavin S staining 6 months post injection. Fyn KO-AAV showed less NFTs than WT-AVV. **g, i** Scale bar: 200 μm. **h** and **i** showed enlarged portions from panels **f** and **g**, respectively; scale bar 50 μm. 3 WT-AAV and 3 Fyn KO-AAV mice were used and representative images were shown. Sections from the same mice were used for panels **a** and **f** and for panels **b** and **g**. While all WT-AAV mice examined contained minimal pathology in the dentate gyrus area, one of three Fyn KO-AAV mice examined had clear Bielschowsky silver stained filaments in the dentate gyrus area and thioflavin S positive staining (**b**, **g**, SI Fig. 4). However, Cook et al., who had also used P0 injection of AAV-tau^P301L^ to create a tauopathy model in WT mice, reported pathology in the dentate gyrus (Fig. 1j in [14]). Therefore, the pathology in the dentate gyrus of the Fyn KO-AAV was not unique to the Fyn KO genotype. The variability between our findings and those of Cook et al. indicated that comparisons would best be made within an experimental system, since between laboratories, the level of tau expression may vary, in part depending on AAV preparation used and virus titer injected

At 6 months of age, WT-AAV mice displayed significant amounts of both Bielschowsky silver stained (Fig. 1a, c) or Thioflavin S positive (Fig. 1f, h) tangles in the CA1–3 region of the hippocampus. In Fyn KO-AAV mice, a reduction in tangle pathology, relative to WT-AAV mice, was observed using Bielschowsky silver staining (Fig. 1b, d) or Thioflavin S (Fig. 1g, i). Upon quantitation of the Bielschowsky silver stain, we found that Fyn depletion resulted in a reduction of 79.6% in tangle pathology relative to WT-AAV (*p* = 0.0187). These results indicated that Fyn played an important role in NFT formation.

**Fig. 2:**
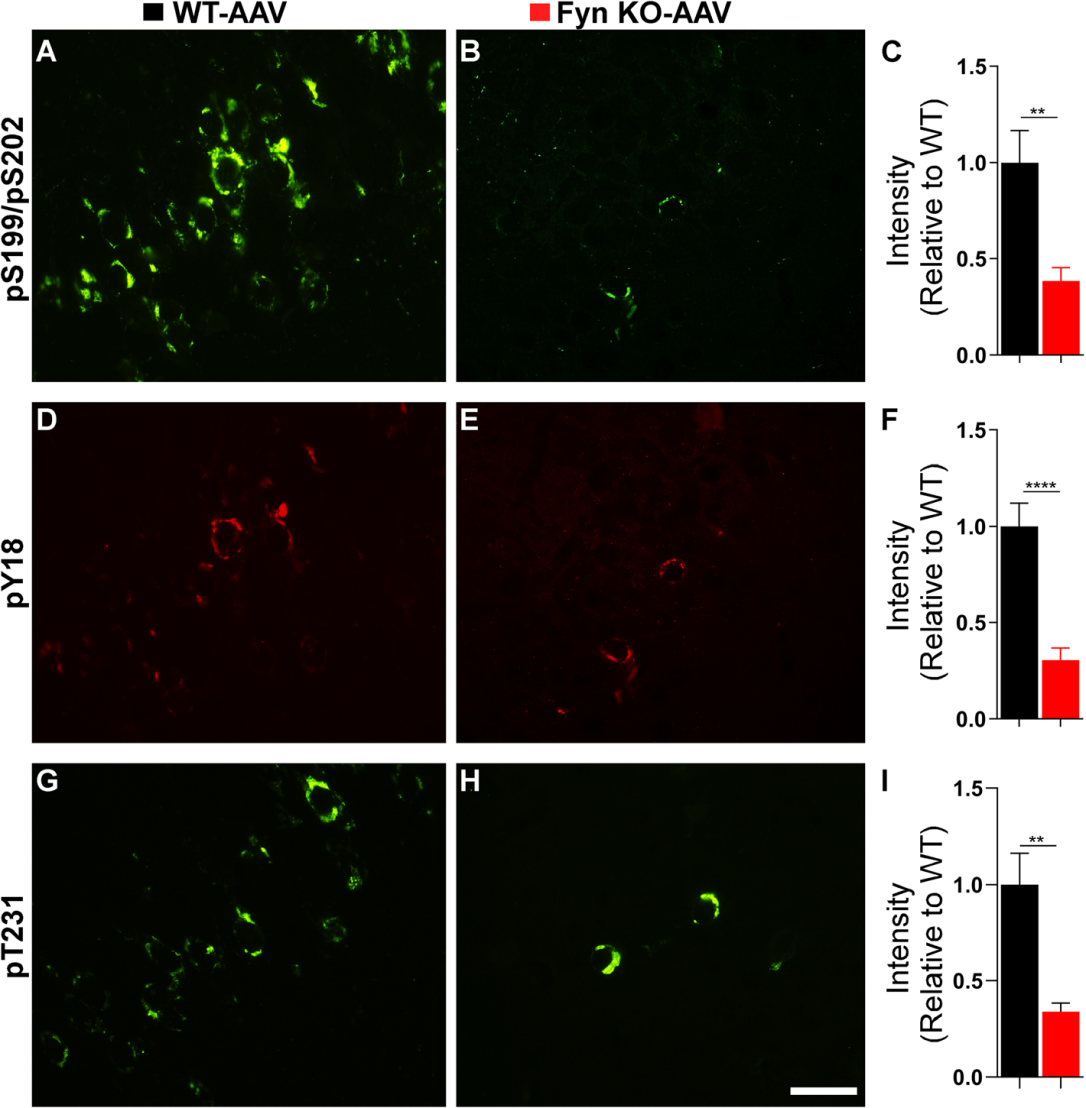
Fyn KO-AAV mice displayed less hyperphosphorylated tau relative to WT-AAV injected mice. Using double immunofluorescent labeling, WT-AAV hippocampus showed robust pS199/pT202 **(a)** and pY18 **(d)** staining of NFTs in the hippocampal CA1 region. Fyn KO-AAV hippocampus showed only limited pS199/pT202 **(b)** and pY18 **(e)** staining of NFTs in the same region. Using adjacent slides from the same mice, staining of pT231 showed robust staining in WT-AAV CA1 **(g)** but fewer NFTS in CA1 of Fyn KO-AAV **(h)**. Intensity was determined as described in Methods (four fields from each of six animals) and p values calculated by t-test. Relative to WT-AAV, Fyn KO-AAV had reduced staining for pS199/pT202 (**c**, *p* = 0.0035), pY18 (**f**, *p* < 0.0001) and pT231 (**i**, *p* = 0.0037). Analysis was carried out using 4 random pictures taken from the hippocampus of 3 WT-AAV and 3 Fyn KO-AAV mice. Representative images were shown. Scale bar: 25 μm.

Given that NFT contain tau species that are abnormally phosphorylated, we examined the effect of Fyn depletion on tau hyperphosphorylation since Fyn is able to both directly and indirectly affect tau phosphorylation. WT-AAV mice displayed strong immunoreactivity to antibodies targeting pS199/pS202 (Fig. 2a), pY18 (Fig. 2d), and pT231 (Fig. 2g) in the hippocampus. In contrast, Fyn KO-AAV mice had a 61.4% reduction of pS199/pS202 (Fig. 2b, c, *p* = 0.0035), 69.5% reduction of pY18 (Fig. 2e, f, *p* < 0.0001), and 66.1% reduction of pT231 (Fig. 2h, i, *p* = 0.0037). Since pY18-tau persisted in Fyn KO-AAV (Fig. 2e), another tyrosine kinase was still acting on tau in the absence of Fyn.

**Fig. 3:**
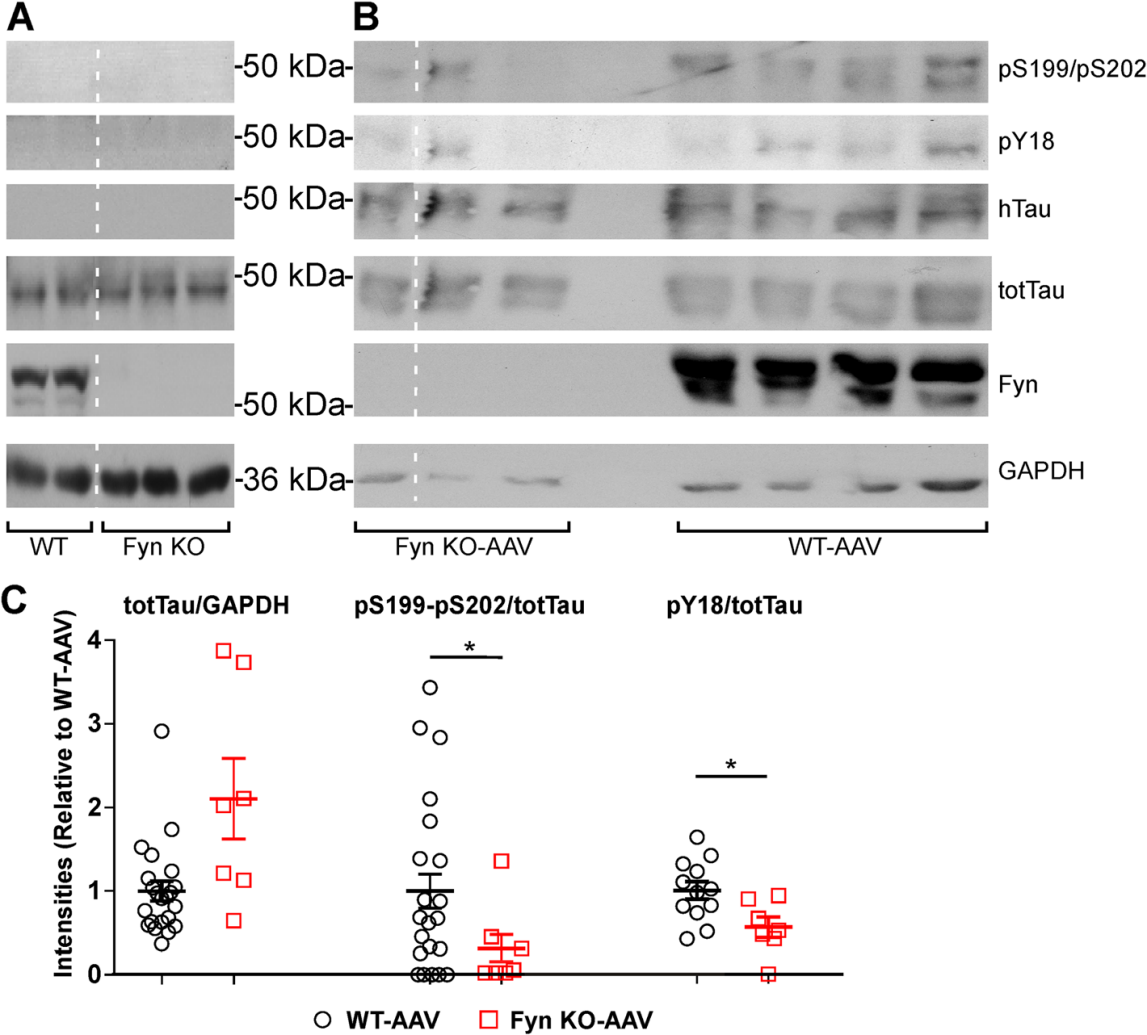
Fyn depletion reduced tau^P301L^-induced abnormal tau hyperphosphorylation. **a** Using soluble brain lysates, uninjected WT and Fyn KO mice did not show any abnormal tau hyperphosphorylation at the pS199/pS202 or pY18 epitopes. (Dotted white lines in A and B indicated places where noncontiguous lanes were brought together from the same blot.) **b** Using soluble brain lysates, WT-AAV and Fyn KO-AAV mice specifically showed the presence of human tau (htau) as well as pS199/pS202 and pY18 epitopes. **c** Quantification for total tau normalized to GAPDH (left), pS199/pS202 normalized to total tau (center), and pY18 normalized to total tau [88]. Relative to WT-AA, Fyn KO-AAV showed decreased tau hyperphosphorylation at pS199/pS202 (19 WT-AAV and 7 Fyn KO-AAV mice used, *p* = 0.0106) and pY18 epitopes (12 WT-AAV and 7 Fyn KO-AAV mice used, *p* = 0.0168) while total tau expression was not significantly different between Fyn KO-AAV and WT-AAV (22 WT-AAV and 7 Fyn KO-AAV mice used, *p* = 0.0532). To calculate *p* values, nonparametric t test was used for total tau and unpaired parametric t-test was used for to pS199/pS202 and pY18

To assess the total level of phosphorylated tau species, soluble lysates from AAV injected WT and Fyn KO brains were subjected to western blot analysis using AT8 (pS199/S202) and pY18 antibodies. Since we had injected AAV-tau^P301L^ by hand, variability in tau expression might arise. To account for this possibility, we also probed the lysates with tau5, an antibody that detected both mouse and human tau, independent of phosphorylation. This allowed us to measure total tau levels for each injected mouse and to normalize pS199/S202 or pY18 levels to total tau levels; a scatter plot was used to display the normalized values. This analysis revealed that Fyn KO-AAV mice had a 72.4% decrease in pS199/pS202 (*p* = 0.0106) and a 44.0% decrease in pY18 levels (*p* = 0.0168) relative to WT-AAV (Fig. 3b, c). Uninjected mice were also examined, but pS199/pS202, pY18, and human tau were not detected (Fig. 3a). The total tau levels between Fyn KO-AAV and WT-AAV were not significantly different (*p* = 0.0532) (Fig. 3b, c).

### Fyn depletion attenuated AAV-tau^P301L^ induced microgliosis

**Fig. 4:**
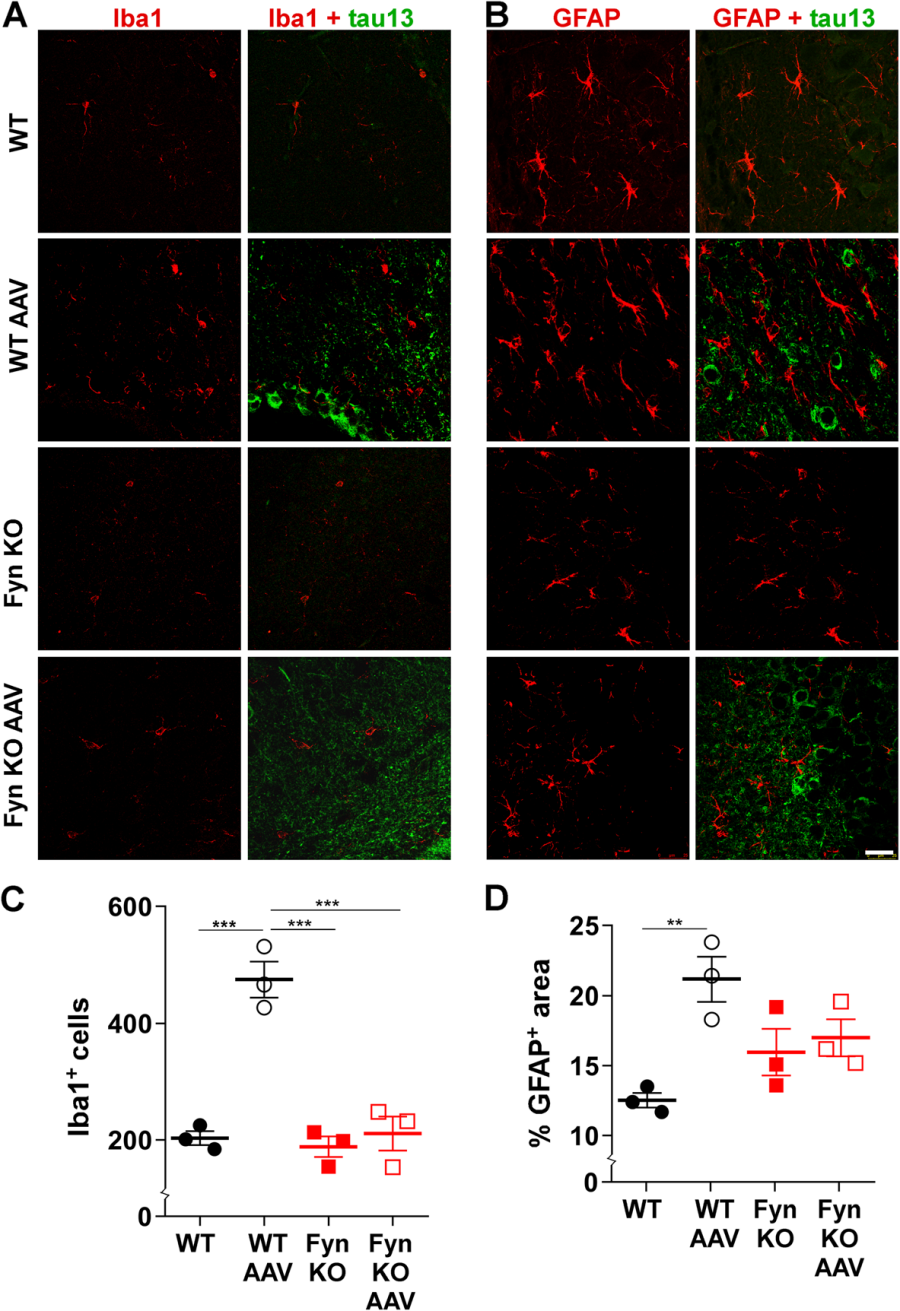
Fyn depletion modulated tau^P301L^-induced microgliosis. **a** WT-AAV had increased total number of Iba1 positive cells in the hippocampus relative to WT uninjected; no such change was found when comparing Fyn KO uninjected and Fyn KO-AAV mice. **b** WT-AAV had increased total area of GFAP positive staining in the hippocampus relative to WT uninjected; no such change was found when comparing Fyn KO uninjected and Fyn KO-AAV mice. **c** In the hippocampus, WT-AAV showed an increase in total number of Iba1 positive cells relative to WT uninjected (*p* = 0.0002) while Fyn KO-AAV did not show any increase relative to Fyn KO uninjected (*p* = 0.9027) or to WT uninjected (*p* = 0.9954). Fyn KO-AAV was significantly less relative to WT-AAV (*p* = 0.0002). **d** In the hippocampus, WT-AAV showed an increase in percent GFAP positive area relative to WT uninjected (*p* = 0.0087) while Fyn KO-AAV did not show any increase relative to Fyn KO uninjected (*p* = 0.9473), WT uninjected (*p* = 0.1712), or WT-AAV (*p* = 0.2115). 3 mice from each group was examined. Scale bar: 25 μm. Two-way ANOVA with Tukey’s post hoc multiple comparisons was used for panels C and D. Statistical results are shown in SI Table 1

Several lines of evidence indicate that tau pathology is associated with inflammation in both human tissues [19, 20, 30] and animal models [3, 45, 88] and that increasing inflammation also worsens tau pathology [34, 38, 51]. Similar to results from Cook et al. [14], we also observed that AAV-tau^P301L^ expression in WT mice caused a significant increase in the number of Iba1 positive microglia in the hippocampus (*p* = 0.0002, Fig. 4a, c). AAV-tau^P301L^ expression also caused an elevated percent area of GFAP positive astrocytes in the same brain regions (*p* = 0.0087, Fig. 4b, d) compared to uninjected WT mice.

Interestingly, Fyn has also been shown to play a role in oligodendrocyte differentiation [77], astrocyte migration [18], and in the production of inflammatory cytokines in natural killer cells [61]. In models of Alzheimer’s disease, Parkinson’s disease, and epilepsy, Fyn has also been implicated in modulating the neuroinflammatory response [55, 57, 70, 80]. We found that while tau^P301L^ increased microgliosis in WT mice, AAV injected Fyn KO mice did not experience tau^P301L^-induced microgliosis when compared to Fyn KO uninjected mice (*p* = 0.9027; Fig. 4a, c). For astrocytosis, a similar result was obtained, where the Fyn KO and Fyn KO-AAV had a similar level of percent GFAP positive area (*p*=0.9473; Fig. 4d).

### Both Fyn depletion and AAV-tau^P301L^ caused behavioral abnormalities

**Fig. 5:**
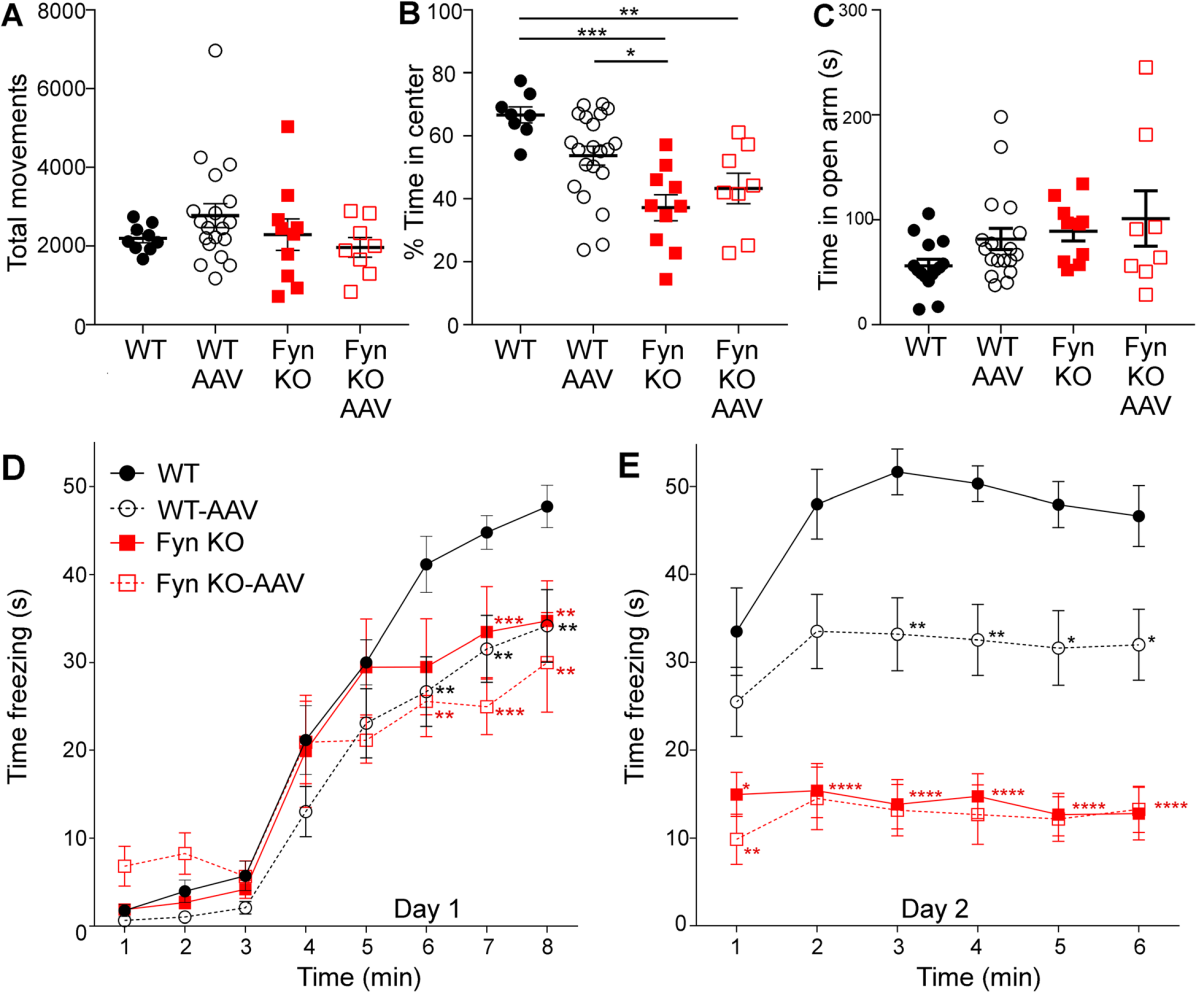
Both Fyn depletion and tau^P301L^ caused behavior abnormality. **a** In the open field test, no difference in the total number of movements was detected between tau^P301L^ injected or uninjected WT and Fyn KO mice (SI Table 1). 8 WT, 21 WT-AAV, 10 Fyn KO, 8 Fyn KO-AAV mice were used. **b** Uninjected WT mice spent more time in the center of the open field apparatus relative to the uninjected Fyn KO and Fyn KO-AAV (*p* = 0.0002 and *p* = 0.0049, respectively). Fyn KO-AAV mice were similar to uninjected Fyn KO (*p* = 0.94). If t-test instead of two-way ANOVA was used, uninjected WT spent significantly more time in the center of the field than WT-AAV (t-test *p* = 0.0333). **c** In the elevated plus maze, the four groups of mice did not behave significantly different, as evaluated by two-way ANOVA (SI Table 1). However, if t-test were used instead, uninjected WT differed significantly from WT-AAV (t-test *p* = 0.0342) whereas uninjected Fyn KO did not differ from Fyn KO-AAV (t-test *p* = 0.7203); uninjected Fyn KO was significantly different from uninjected WT (t-test *p* = 0.0022). 15 WT, 18 WT-AAV, 10 Fyn KO, and 8 Fyn KO-AAV were used. **d** Relative to uninjected WT, WT-AAV mice had learning deficits during training day of contextual fear conditioning (day 1). Relative to uninjected Fyn KO, Fyn KO-AAV mice did not have any learning disabilities. Asterisks indicate statistical comparisons to WT uninjected mice. 12 WT, 20 WT-AAV, 8 Fyn KO, and 9 Fyn KO-AAV were used. **e** Relative to uninjected WT, WT-AAV mice displayed memory deficits in the testing day of contextual fear conditioning (day 2) while Fyn KO-AAV mice did not, relative to Fyn KO uninjected mice. However, both Fyn KO and Fyn KO-AAV displayed memory deficits relative to WT uninjected and WT-AAV mice. Asterisks indicate comparisons to WT uninjected mice; Fyn KO and Fyn KO-AAV mice shared the same p-values when compared to WT. Significant differences were found between WT-AAV and Fyn KO/Fyn KO-AAV at times 2,3,4,5, and 6 min (*p* values are in SI Table 1). For **d** and **e**, * denotes *p* ≤ 0.05, ** denotes *p* ≤ 0.01, *** denotes *p* ≤ 0.001, ****denotes *p* ≤ 0.0001. Two-way ANOVA with Tukey’s post hoc multiple comparisons were used for all panels. Statistical results are shown in SI Table 1

To determine how Fyn depletion impacted tau^P301L^-induced behavioral abnormalities, we evaluated performance on tasks designed to assess learning and memory as well as anxiety, which were all clinical manifestations of FTDP-17. In the open field assay, although none of the mice experienced significant differences in total movement (Fig. 5a), uninjected Fyn KO mice spent less time in the center of the apparatus compared to uninjected WT mice (*p* = 0.0002; Fig. 5b), indicating an anxiety phenotype. In the elevated plus maze, none of the mice experienced significant differences in time spent in the open arms (Fig. 5c). However, we did note that if the comparison was restricted to just the two groups, using parametric t test (SI Table 1), WT-AAV injected mice behaved significantly differently, relative to WT mice, in both the open field assay (*p* = 0.0333, Fig. 5b) and the elevated plus maze (*p* = 0.0342, Fig. 5c). Such a result agreed with data obtained by Cook and colleagues who used both the p0 AAV-tau^P301L^ approach [14], and the rTg4510 tauopathy mouse model [13]. In addition, we also noted that relative to uninjected WT mice, uninjected Fyn KO mice also behaved differently in elevated plus maze (*p* = 0.0022, SI Table 1) if the comparison was restricted to just the two groups, using parametric t test.

In contextual fear conditioning, WT-AAV injected mice, relative to WT uninjected mice showed significant learning deficits during acquisition of shock on training day (day 1) (Fig. 5d) and significant memory deficit during the recall of shock in context on testing day (day 2) (Fig. 5e), as analyzed with two-way ANOVA. Interestingly, besides having a role in mediating anxiety behaviors (Fig. 5b), Fyn is also known to play a role in LTP and memory formation, which is consistent with our finding that uninjected Fyn KO mice displayed significant learning and memory deficits in contextual fear conditioning relative to WT uninjected mice (Fig. 5d, e). However, unlike WT-AAV, Fyn KO-AAV mice did not exhibit any learning (Fig. 5D) or memory deficits (Fig. 5e) relative to its uninjected mice. This agreed with our finding that Fyn KO-AAV mice did not experience any abnormalities in anxiety behaviors relative to Fyn KO uninjected mice (Fig. 5b, c). Lastly, we noted that the deficits exhibited by the uninjected Fyn KO mice were either similar to (Fig. 5c, d) or larger (Fig. 5b, e) than those exhibited by WT-AAV.

### Fyn depletion prevented tau^P301L^ induced synaptic protein degradation while no neuronal cell loss was detected in AAV injected mice

**Fig. 6:**
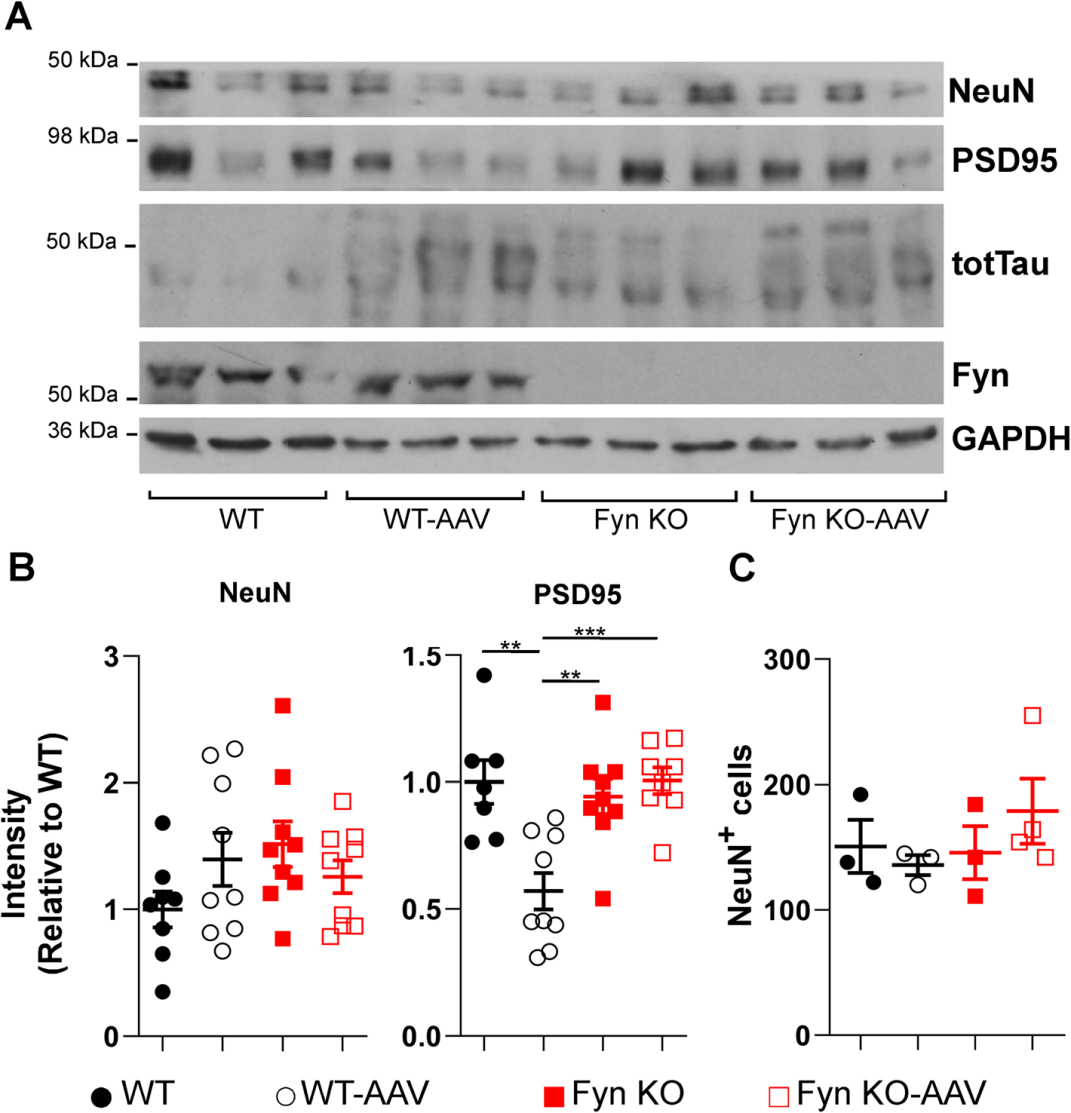
AAV-tau^P301L^ did not cause overt neuronal cell loss at 6 months of age. **a** Crude brain lysates, probed by western blot, showed no change in NeuN between all mice groups. However, PSD95 was significantly decreased in WT-AAV mice. **b** Left: Quantification of NeuN showed no difference between the 4 groups (SI Table 1). Right: Quantification of PSD95 showed a 39.1% decrease in PSD95 in WT-AAV relative to WT uninjected (*p* = 0.0049). There was no difference between WT uninjected and Fyn KO uninjected mice (*p* = 0.9999) nor between WT uninjected and Fyn KO-AAV (*p* = 0.9073). For NeuN, 8 WT, 9 WT-AAV, 9 Fyn KO, and 9 Fyn KO-AAV were used. For PSD95, 7 WT, 9 WT-AAV, 9 Fyn KO, and 8 Fyn KO-AAV were used. **c**) Quantification of NeuN positive cells showed no difference between the four groups in Ammon’s horn (*p* = 0.5214). 3 WT, 3 WT-AAV, 3 Fyn KO, and 4 Fyn KO-AAV mice were examined. Two-way ANOVA with Tukey’s post hoc multiple comparisons were used for all panels. Statistical results are shown in SI Table 1

To determine whether the behavioral deficits experienced by the WT-AAV mice were due to overt neuronal loss or more subtle synaptic abnormalities, we first probed whole brain crude lysates for NeuN, as a marker for neurons, and for PSD95, as a synaptic marker, in WT, WT-AAV, Fyn KO, and Fyn KO-AAV mice. In the crude brain lysates, there was no statistical difference in NeuN for all four groups of mice (Fig. 6 A, B left, SI Table 1). However, we found that in PSD95, WT-AAV had a 39.1% reduction relative to WT uninjected mice (*p* = 0.0049), a 39.6% reduction relative to Fyn KO uninjected (*p* = 0.0021), and a 43.3% reduction relative to Fyn KO-AAV (*p* = 0.00005; Fig. 6a, b right). There was no difference between WT uninjected and Fyn KO uninjected mice (*p* = 0.9999), between WT uninjected and Fyn KO-AAV (*p* = 0.9073), or between Fyn KO and Fyn KO-AAV (*p* = 0.9162; Fig. 6a, b right). We also examined brain slices stained with anti-NeuN and found no differences between the different groups in the total number of neurons in the Ammon’s horn of the hippocampus (Fig. 6c, SI Table 1).

**Fig. 7:**
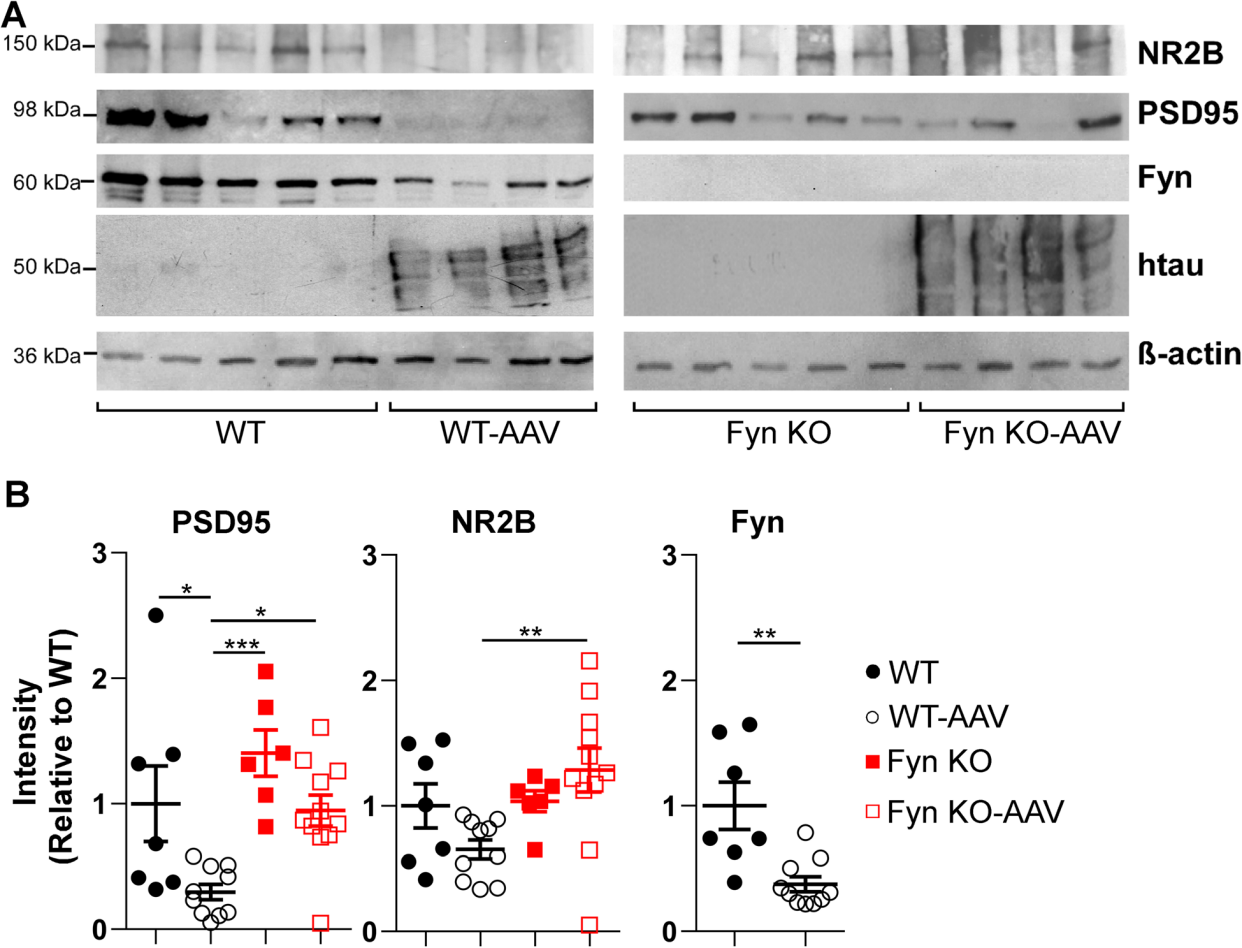
Fyn depletion prevented tau^P301L^ induced synaptic protein degradation. **a** Synaptosome preps from WT and Fyn KO uninjected mice, WT-AAV, and Fyn KO-AAV mice were probed for NR2B, PSD95, and Fyn. 7 WT, 10 WT-AAV, 6 Fyn KO, and 11 Fyn KO-AAV were used. **b** Compared to WT uninjected mice, WT-AAV had significantly decreased concentrations of PSD95, (*p* = 0.027) and Fyn (*p* = 0.0024); using t-test, a decreased concentration of NR2B (*p* = 0.0304), was also observed. In contrast, relative to Fyn KO uninjected mice, Fyn KO-AAV had no difference in the concentrations of PSD95 (*p* = 0.2519) or NR2B (*p* = 0.6533). There was also no difference between WT and Fyn KO uninjected mice for PSD95 (*p* = 0.4378) and NR2B (*p* = 0.9986). Two-way ANOVA with Tukey’s post hoc multiple comparisons were used for PSD95 and NR2B panels. T-test was used for Fyn. Statistical results are shown in SI Table 1

To further characterize the synaptic integrity of the mice, we obtained crude synaptosome preparations from WT, WT-AAV, Fyn KO, and Fyn KO-AAV brains and probed for postsynaptic markers such as PSD 95 and NR2B. Again, we found that relative to WT uninjected mice, WT-AAV mice had a 70.3% decrease in PSD95 (*p* = 0.027) (Fig. 7a, b). The 34.3% decrease in NR2B levels was not significant by two-way ANOVA (*p* = 0.3607) but was significant if only WT and WT-AAV were compared using the unpaired t-test (*p* = 0.0304, SI Table 1). Similar to our previous findings [46], Fyn KO uninjected animals had no changes in PSD95 (*p* = 0.4378) or NR2B (*p* = 0.9986) when compared to WT uninjected animals (Fig. 7a,b; SI Table 1), indicating that Fyn depletion itself did not cause alterations in synaptic integrity at 6 months of age. When examining the Fyn KO-AAV mice, we found that the levels of PSD95 and NR2B in Fyn KO-AAV mice were comparable to their levels in either WT uninjected mice or Fyn KO uninjected mice (Fig. 7b, 5B; SI Table 1). Therefore, our data showed that Fyn depletion was able to reverse tau^P301L^ induced synaptic loss. Interestingly, the WT-AAV synaptosome preps also had a 62.5% decrease in Fyn when compared to uninjected WT mice (Fig. 7a, b) (*p* = 0.0024), an effect that may be due to the overall loss of PSD protein. Our data supports findings by other investigators where mice expressing tau^P301L^ at 6 months of age displayed synaptic loss without overt neuronal loss [14, 16, 64, 69].

## Discussion

In this study, we reproduced the P301L tauopathy mouse model using injection of AAV-tau^P301L^ into P0 WT mouse brain, as previously described [14], then used the same strategy to investigate the role of Fyn by injecting Fyn KO P0 brains. In the WT mice, we reproduced the results of Cook et al., where widespread expression of human tau was reported, together with tau hyperphosphorylation, neurofibrillary tangles, inflammation, behavioral abnormalities, and loss of postsynaptic markers [14]. In working with homozygous Fyn KO mice, one caveat was their tendency to develop non-obstructive hydrocephalus [22]. In our study, given that WT-AAV injected mice did not develop hydrocephalus, AAV-tau^P301L^ injection did not correlate with the occurrence of hydrocephalus. However, the combination of the P0 brain injection and the homozygous Fyn KO trait resulted in a higher incidence of hydrocephalus. Nevertheless, by eliminating mice with severe hydrocephalus, we were able to show that depleting Fyn (1) reduced tau^P301L^-induced abnormal tau hyperphosphorylation and NFT formation and (2) prevented tau^P301L^-induced microgliosis and synaptic loss. These data provide evidence for Fyn as an essential protein involved in the disease pathogenesis of tauopathies.

In terms of regulating tau phosphorylation at Y18, Fyn depletion reduced but did not eliminate pY18. Since our lab has found that both Fyn and Src can phosphorylate this residue [42], Src may phosphorylate Y18 in a Fyn KO. Moreover, other tyrosine kinases such as Syk [40] and Lck [68] have also been reported to phosphorylate Y18. This suggests that the phosphorylation of Y18 is sufficiently important such that in spite of the absence of Fyn, the residue is phosphorylated. The importance of pY18-tau for glutamate-induced calcium influx and excitotoxicity in neurons has been shown [54]. As for ser/thr phosphorylation, we found that depleting Fyn caused a significant reduction in phosphorylation of tau at a Cdk5 site (S199/S202) and a GSK3β site (T231), suggesting that depletion of Fyn may have resulted in the reduction of Cdk5 and GSK3β activities. In the Fyn KO, the reduction in tau hyperphosphorylation correlated with a reduction in Bielschowsky silver stained and thioflavin S positive NFT. While our findings did not rule out effects caused by potential strain differences, our data suggested that Fyn is critical for NFT formation. 

In our somatic transgenic model, we were also able to replicate the neuroinflammatory changes, such as the prominent microgliosis and astrocytosis associated with tau^P301L^ expression [14, 28, 45, 51]. Our data showed that Fyn depletion was able to eliminate the effects of AAV-tau^p301L^ injection on microgliosis and astrocytosis, providing evidence that Fyn plays an important role in the microglial inflammatory response in tauopathies.

Lastly, the impact of Fyn depletion on tau^P301L^-induced behavioral deficits was investigated. Our WT-AAV injected mice developed aberrant exploratory behavior and cognitive deficits that were also found in other transgenic FTDP-17 tauopathy mouse models [62]. At 6 months of age, these deficits have been attributed to tau^P301L^-induced changes in dendritic spine architecture, synaptic dysfunction and loss, and abnormalities in electrophysiology, all preceding NFT deposition and neuronal loss in the rTg4510 transgenic FTDP tauopathy mouse model [14, 16, 64]. Our findings are consistent, as our data also showed losses in postsynaptic proteins without neuronal cell loss in WT-AAV.

In analyzing the behavioral deficits of Fyn KO-AAV, we found that the introduction of tau^P301L^ was not able to induce further deficits in the behaviors of Fyn KO mice (Fig. 5). However, Fyn KO mice were already known to have abnormal exploratory behavior and cognitive deficits relative to WT mice [31, 46, 53], stemming from Fyn’s role in mediating LTP and memory formation through the phosphorylation of NMDARs [23, 35, 56, 83]. Therefore, the Fyn KO mice may already be performing at such a low level that further decreases might not be possible no matter the insult, such as tau^P301L^ (also known as the “floor effect”). Moreover, it was difficult to determine whether tau^P301L^ required Fyn to induce behavior abnormalities or that the deficits caused by Fyn depletion alone superseded those induced by tau^P301L^. The latter possibility (the “floor effect”) was supported by our finding that in two tests, the Fyn KO mice had significantly more deficits than the WT-AAV (Fig. 5b, e, SI Table 1). The fact that Fyn KO and Fyn KO-AAV both had normal levels of PSD95 and NR2B (Figs. 6 and 7) suggested that Fyn KO had no synapse loss and that tau^P301L^ could not induce synapse loss in the absence of Fyn. These findings suggest that synapse loss cannot be used to explain the behavioral deficits of the Fyn KO and Fyn KO-AAV mice.

In summary, using AAV to create a P301L tauopathy mouse model on a Fyn KO background, we have found that Fyn was critical for neurofibrillary tangle formation and tau hyperphosphorylation. Our data suggests that reducing Fyn activity might be an effective therapy for tauopathies. However, given the importance of Fyn in normal physiological processes, such therapy would be difficult, especially since dementia patients usually have reduced cognitive reserves. Thus, more specific therapies aimed at targeting either disease related processes involving Fyn or Fyn interaction partners may yield a more desirable therapeutic result; the recent report of a peptide inhibitor of the tau-Fyn interaction [65] is a first step in this direction. In closing, as Aβ oligomers had no role in these findings, Fyn alone has an important role in tauopathy.

## Supplementary information

**Additional file 1. Supplementary Information**

## Data Availability

All data used/analyzed during the current study are available from the corresponding author on reasonable request.

## References

[1] Ballatore C, Lee VM, Trojanowski JQ (2007). Tau-mediated neurodegeneration in Alzheimer's disease and related disorders. Nat Rev Neurosci.

[2] Bhaskar K, Hobbs GA, Yen SH, Lee G (2010). Tyrosine phosphorylation of tau accompanies disease progression in transgenic mouse models of tauopathy. Neuropathol Appl Neurobiol.

[3] Bhaskar K, Konerth M, Kokiko-Cochran ON, Cardona A, Ransohoff RM, Lamb BT (2010). Regulation of tau pathology by the microglial fractalkine receptor. Neuron.

[4] Bhaskar K, Yen SH, Lee G (2005). Disease-related modifications in tau affect the interaction between Fyn and tau. J Biol Chem.

[5] Boehm J (2013). A 'danse macabre': tau and Fyn in STEP with amyloid beta to facilitate induction of synaptic depression and excitotoxicity. Eur J Neurosci.

[6] Braithwaite SP, Adkisson M, Leung J, Nava A, Masterson B, Urfer R, Oksenberg D, Nikolich K (2006). Regulation of NMDA receptor trafficking and function by striatal-enriched tyrosine phosphatase (STEP). Eur J Neurosci.

[7] Brandt R, Leger J, Lee G (1995). Interaction of tau with the neural plasma membrane mediated by tau's amino-terminal projection domain. J Cell Biol.

[8] Carter CS, Vogel TW, Zhang Q, Seo S, Swiderski RE, Cassell MD, Thedens DR, Keppler-Noreuil KM, Nopoulos P, Moninger TO (2012). Abnormal development of NG2^+^PDGFRα^+^ neural progenitor cells leads to neonatal hydrocephalus in a ciliopathy mouse model. Nat Med.

[9] Chakrabarty P, Rosario A, Cruz P, Siemienski Z, Ceballos-Diaz C, Crosby K, Jansen K, Borchelt DR, Kim JY, Jankowsky JL (2013). Capsid serotype and timing of injection determines AAV transduction in the neonatal mice brain. PLoS One.

[10] Chin J, Palop JJ, Puolivali J, Massaro C, Bien-Ly N, Gerstein H, Scearce-Levie K, Masliah E, Mucke L (2005). Fyn kinase induces synaptic and cognitive impairments in a transgenic mouse model of Alzheimer's disease. J Neurosci.

[11] Chin J, Palop JJ, Yu GQ, Kojima N, Masliah E, Mucke L (2004). Fyn kinase modulates synaptotoxicity, but not aberrant sprouting, in human amyloid precursor protein transgenic mice. J Neurosci.

[12] Clark LN, Poorkaj P, Wszolek Z, Geschwind DH, Nasreddine ZS, Miller B, Li D, Payami H, Awert F, Markopoulou K (1998). Pathogenic implications of mutations in the tau gene in pallido-ponto-nigral degeneration and related neurodegenerative disorders linked to chromosome 17. Proc Natl Acad Sci U S A.

[13] Cook C, Dunmore JH, Murray ME, Scheffel K, Shukoor N, Tong J, Castanedes-Casey M, Phillips V, Rousseau L, Penuliar MS (2014). Severe amygdala dysfunction in a MAPT transgenic mouse model of frontotemporal dementia. Neurobiol Aging.

[14] Cook C, Kang SS, Carlomagno Y, Lin WL, Yue M, Kurti A, Shinohara M, Jansen-West K, Perkerson E, Castanedes-Casey M (2015). Tau deposition drives neuropathological, inflammatory and behavioral abnormalities independently of neuronal loss in a novel mouse model. Hum Mol Genet.

[15] Coryell MW, Ziemann AE, Westmoreland PJ, Haenfler JM, Kurjakovic Z, Zha XM, Price M, Schnizler MK, Wemmie JA (2007). Targeting ASIC1a reduces innate fear and alters neuronal activity in the fear circuit. Biol Psychiatry.

[16] Crimins JL, Rocher AB, Luebke JI (2012). Electrophysiological changes precede morphological changes to frontal cortical pyramidal neurons in the rTg4510 mouse model of progressive tauopathy. Acta Neuropathol.

[17] Dehmelt L, Halpain S (2005). The MAP 2/tau family of microtubule-associated proteins. Genome Biol.

[18] Dey N, Crosswell HE, De P, Parsons R, Peng Q, Su JD, Durden DL (2008). The protein phosphatase activity of PTEN regulates SRC family kinases and controls glioma migration. Cancer Res.

[19] Gebicke-Haerter PJ (2001). Microglia in neurodegeneration: molecular aspects. Microsc Res Tech.

[20] Gerhard A, Pavese N, Hotton G, Turkheimer F, Es M, Hammers A, Eggert K, Oertel W, Banati RB, Brooks DJ (2006). In vivo imaging of microglial activation with [11C](R)-PK11195 PET in idiopathic Parkinson's disease. Neurobiol Dis.

[21] Goedert M, Eisenberg DS, Crowther RA (2017). Propagation of tau aggregates and neurodegeneration. Annu Rev Neurosci.

[22] Goto J, Tezuka T, Nakazawa T, Sagara H, Yamamoto T (2008). Loss of Fyn tyrosine kinase on the C57BL/6 genetic background causes hydrocephalus with defects in oligodendrocyte development. Mol Cell Neurosci.

[23] Grant SG, O'Dell TJ, Karl KA, Stein PL, Soriano P, Kandel ER (1992). Impaired long-term potentiation, spatial learning, and hippocampal development in fyn mutant mice. Science.

[24] Hanger DP, Hughes K, Woodgett JR, Brion JP, Anderton BH (1992). Glycogen synthase kinase-3 induces Alzheimer's disease-like phosphorylation of tau: generation of paired helical filament epitopes and neuronal localisation of the kinase. Neurosci Lett.

[25] Hashiguchi M, Saito T, Hisanaga S, Hashiguchi T (2002). Truncation of CDK5 activator p35 induces intensive phosphorylation of Ser202/Thr205 of human tau. J Biol Chem.

[26] Ho GJ, Hashimoto M, Adame A, Izu M, Alford MF, Thal LJ, Hansen LA, Masliah E (2005). Altered p59Fyn kinase expression accompanies disease progression in Alzheimer's disease: implications for its functional role. Neurobiol Aging.

[27] Hoover BR, Reed MN, Su J, Penrod RD, Kotilinek LA, Grant MK, Pitstick R, Carlson GA, Lanier LM, Yuan LL (2010). Tau mislocalization to dendritic spines mediates synaptic dysfunction independently of neurodegeneration. Neuron.

[28] Hunsberger HC, Rudy CC, Weitzner DS, Zhang C, Tosto DE, Knowlan K, Xu Y, Reed MN (2014). Effect size of memory deficits in mice with adult-onset P301L tau expression. Behav Brain Res.

[29] Hutton M, Lendon CL, Rizzu P, Baker M, Froelich S, Houlden H, Pickering-Brown S, Chakraverty S, Isaacs A, Grover A (1998). Association of missense and 5′-splice-site mutations in tau with the inherited dementia FTDP-17. Nature.

[30] Ishizawa K, Dickson DW (2001). Microglial activation parallels system degeneration in progressive supranuclear palsy and corticobasal degeneration. J Neuropathol Exp Neurol.

[31] Isosaka T, Hattori K, Kida S, Kohno T, Nakazawa T, Yamamoto T, Yagi T, Yuasa S (2008). Activation of Fyn tyrosine kinase in the mouse dorsal hippocampus is essential for contextual fear conditioning. Eur J Neurosci.

[32] Ittner LM, Ke YD, Delerue F, Bi M, Gladbach A, van Eersel J, Wölfing H, Chieng BC, Christie MJ, Napier IA (2010). Dendritic function of tau mediates amyloid-β toxicity in Alzheimer's disease mouse models. Cell.

[33] Kaufman AC, Salazar SV, Haas LT, Yang J, Kostylev MA, Jeng AT, Robinson SA, Gunther EC, van Dyck CH, Nygaard HB (2015). Fyn inhibition rescues established memory and synapse loss in Alzheimer mice. Ann Neurol.

[34] Kitazawa M, Oddo S, Yamasaki TR, Green KN, LaFerla FM (2005). Lipopolysaccharide-induced inflammation exacerbates tau pathology by a cyclin-dependent kinase 5-mediated pathway in a transgenic model of Alzheimer's disease. J Neurosci.

[35] Kojima N, Wang J, Mansuy IM, Grant SG, Mayford M, Kandel ER (1997). Rescuing impairment of long-term potentiation in Fyn-deficient mice by introducing Fyn transgene. Proc Natl Acad Sci U S A.

[36] Kolarova M, Garcia-Sierra F, Bartos A, Ricny J, Ripova D (2012). Structure and pathology of tau protein in Alzheimer disease. Int J Alzheimers Dis.

[37] Kopeikina KJ, Polydoro M, Tai HC, Yaeger E, Carlson GA, Pitstick R, Hyman BT, Spires-Jones TL (2013). Synaptic alterations in the rTg4510 mouse model of tauopathy. J Comp Neurol.

[38] Krstic D, Madhusudan A, Doehner J, Vogel P, Notter T, Imhof C, Manalastas A, Hilfiker M, Pfister S, Schwerdel C (2012). Systemic immune challenges trigger and drive Alzheimer-like neuropathology in mice. J Neuroinflammation.

[39] Lambert MP, Barlow AK, Chromy BA, Edwards C, Freed R, Liosatos M, Morgan TE, Rozovsky I, Trommer B, Viola KL (1998). Diffusible, nonfibrillar ligands derived from Aβ_1-42_ are potent central nervous system neurotoxins. Proc Natl Acad Sci U S A.

[40] Lebouvier T, Scales TM, Williamson R, Noble W, Duyckaerts C, Hanger DP, Reynolds CH, Anderton BH, Derkinderen P (2009). The microtubule-associated protein tau is also phosphorylated on tyrosine. J Alzheimers Dis.

[41] Lee G, Newman ST, Gard DL, Band H, Panchamoorthy G (1998). Tau interacts with src-family non-receptor tyrosine kinases. J Cell Sci.

[42] Lee G, Thangavel R, Sharma VM, Litersky JM, Bhaskar K, Fang SM, Do LH, Andreadis A, Van Hoesen G, Ksiezak-Reding H (2004). Phosphorylation of tau by fyn: implications for Alzheimer's disease. J Neurosci.

[43] Lesort M, Jope RS, Johnson GV (1999). Insulin transiently increases tau phosphorylation: involvement of glycogen synthase kinase-3β and Fyn tyrosine kinase. J Neurochem.

[44] Leugers CJ, Lee G (2010). Tau potentiates nerve growth factor-induced mitogen-activated protein kinase signaling and neurite initiation without a requirement for microtubule binding. J Biol Chem.

[45] Lewis J, McGowan E, Rockwood J, Melrose H, Nacharaju P, Van Slegtenhorst M, Gwinn-Hardy K, Paul Murphy M, Baker M, Yu X (2000). Neurofibrillary tangles, amyotrophy and progressive motor disturbance in mice expressing mutant (P301L) tau protein. Nat Genet.

[46] Liu G, Thangavel R, Rysted J, Kim Y, Francis MB, Adams E, Lin Z, Taugher RJ, Wemmie JA, Usachev YM (2019). Loss of tau and Fyn reduces compensatory effects of MAP 2 for tau and reveals a Fyn-independent effect of tau on calcium. J Neurosci Res.

[47] Lund ET, McKenna R, Evans DB, Sharma SK, Mathews WR (2001). Characterization of the in vitro phosphorylation of human tau by tau protein kinase II (cdk5/p20) using mass spectrometry. J Neurochem.

[48] Luo YQ, Hirashima N, Li YH, Alkon DL, Sunderland T, Etcheberrigaray R, Wolozin B (1995). Physiological levels of beta-amyloid increase tyrosine phosphorylation and cytosolic calcium. Brain Res.

[49] Mandelkow EM, Drewes G, Biernat J, Gustke N, Van Lint J, Vandenheede JR, Mandelkow E (1992). Glycogen synthase kinase-3 and the Alzheimer-like state of microtubule-associated protein tau. FEBS Lett.

[50] Mandelkow EM, Mandelkow E (2012). Biochemistry and cell biology of tau protein in neurofibrillary degeneration. Cold Spring Harb Perspect Med.

[51] Maphis N, Xu G, Kokiko-Cochran ON, Jiang S, Cardona A, Ransohoff RM, Lamb BT, Bhaskar K (2015). Reactive microglia drive tau pathology and contribute to the spreading of pathological tau in the brain. Brain.

[52] Martin L, Latypova X, Terro F (2011). Post-translational modifications of tau protein: implications for Alzheimer's disease. Neurochem Int.

[53] Miyakawa T, Yagi T, Kagiyama A, Niki H (1996). Radial maze performance, open-field and elevated plus-maze behaviors in Fyn-kinase deficient mice: further evidence for increased fearfulness. Brain Res Mol Brain Res.

[54] Miyamoto T, Stein L, Thomas R, Djukic B, Taneja P, Knox J, Vossel K, Mucke L (2017). Phosphorylation of tau at Y18, but not tau-fyn binding, is required for tau to modulate NMDA receptor-dependent excitotoxicity in primary neuronal culture. Mol Neurodegener.

[55] Moore KJ, El Khoury J, Medeiros LA, Terada K, Geula C, Luster AD, Freeman MW (2002). A CD36-initiated signaling cascade mediates inflammatory effects of beta-amyloid. J Biol Chem.

[56] Nakazawa T, Komai S, Tezuka T, Hisatsune C, Umemori H, Semba K, Mishina M, Manabe T, Yamamoto T (2001). Characterization of Fyn-mediated tyrosine phosphorylation sites on GluR epsilon 2 (NR2B) subunit of the N-methyl-D-aspartate receptor. J Biol Chem.

[57] Panicker N, Saminathan H, Jin H, Neal M, Harischandra DS, Gordon R, Kanthasamy K, Lawana V, Sarkar S, Luo J (2015). Fyn kinase regulates microglial Neuroinflammatory responses in cell culture and animal models of Parkinson's disease. J Neurosci.

[58] Patrick GN, Zukerberg L, Nikolic M, de la Monte S, Dikkes P, Tsai LH (1999). Conversion of p35 to p25 deregulates Cdk5 activity and promotes neurodegeneration. Nature.

[59] Polydoro M, Dzhala VI, Pooler AM, Nicholls SB, McKinney AP, Sanchez L, Pitstick R, Carlson GA, Staley KJ, Spires-Jones TL (2014). Soluble pathological tau in the entorhinal cortex leads to presynaptic deficits in an early Alzheimer's disease model. Acta Neuropathol.

[60] Poorkaj P, Bird TD, Wijsman E, Nemens E, Garruto RM, Anderson L, Andreadis A, Wiederholt WC, Raskind M, Schellenberg GD (1998). Tau is a candidate gene for chromosome 17 frontotemporal dementia. Ann Neurol.

[61] Rajasekaran K, Kumar P, Schuldt KM, Peterson EJ, Vanhaesebroeck B, Dixit V, Thakar MS, Malarkannan S (2013). Signaling by Fyn-ADAP via the Carma1-Bcl-10-MAP 3K7 signalosome exclusively regulates inflammatory cytokine production in NK cells. Nat Immunol.

[62] Ramsden M, Kotilinek L, Forster C, Paulson J, McGowan E, SantaCruz K, Guimaraes A, Yue M, Lewis J, Carlson G (2005). Age-dependent neurofibrillary tangle formation, neuron loss, and memory impairment in a mouse model of human tauopathy (P301L). J Neurosci.

[63] Roche KW, Standley S, McCallum J, Dune Ly C, Ehlers MD, Wenthold RJ (2001). Molecular determinants of NMDA receptor internalization. Nat Neurosci.

[64] Rocher AB, Crimins JL, Amatrudo JM, Kinson MS, Todd-Brown MA, Lewis J, Luebke JI (2010). Structural and functional changes in tau mutant mice neurons are not linked to the presence of NFTs. Exp Neurol.

[65] Rush T, Roth JR, Thompson SJ, Aldaher AR, Cochran JN, Roberson ED (2020). A peptide inhibitor of tau-SH3 interactions ameliorates amyloid-beta toxicity. Neurobiol Dis.

[66] Sahara N, Murayama M, Higuchi M, Suhara T, Takashima A (2014). Biochemical distribution of tau protein in synaptosomal fraction of transgenic mice expressing human P301L tau. Front Neurol.

[67] Sasaki Y, Cheng C, Uchida Y, Nakajima O, Ohshima T, Yagi T, Taniguchi M, Nakayama T, Kishida R, Kudo Y (2002). Fyn and Cdk5 mediate semaphorin-3A signaling, which is involved in regulation of dendrite orientation in cerebral cortex. Neuron.

[68] Scales TM, Derkinderen P, Leung KY, Byers HL, Ward MA, Price C, Bird IN, Perera T, Kellie S, Williamson R (2011). Tyrosine phosphorylation of tau by the SRC family kinases lck and fyn. Mol Neurodegener.

[69] Schindowski K, Bretteville A, Leroy K, Begard S, Brion JP, Hamdane M, Buee L (2006). Alzheimer's disease-like tau neuropathology leads to memory deficits and loss of functional synapses in a novel mutated tau transgenic mouse without any motor deficits. Am J Pathol.

[70] Sharma S, Carlson S, Puttachary S, Sarkar S, Showman L, Putra M, Kanthasamy AG, Thippeswamy T (2018). Role of the Fyn-PKCdelta signaling in SE-induced neuroinflammation and epileptogenesis in experimental models of temporal lobe epilepsy. Neurobiol Dis.

[71] Sharma VM, Litersky JM, Bhaskar K, Lee G (2007). Tau impacts on growth-factor-stimulated actin remodeling. J Cell Sci.

[72] Shirazi SK, Wood JG (1993). The protein tyrosine kinase, fyn, in Alzheimer's disease pathology. Neuroreport.

[73] Simic G, Babic Leko M, Wray S, Harrington C, Delalle I, Jovanov-Milosevic N, Bazadona D, Buee L, de Silva R, Di Giovanni G (2016). Tau protein hyperphosphorylation and aggregation in Alzheimer's disease and other tauopathies, and possible neuroprotective strategies. Biomolecules.

[74] Singh-Bains MK, Linke V, Austria MDR, Tan AYS, Scotter EL, Mehrabi NF, Faull RLM, Dragunow M (2019). Altered microglia and neurovasculature in the Alzheimer's disease cerebellum. Neurobiol Dis.

[75] Snyder EM, Nong Y, Almeida CG, Paul S, Moran T, Choi EY, Nairn AC, Salter MW, Lombroso PJ, Gouras GK (2005). Regulation of NMDA receptor trafficking by amyloid-β. Nat Neurosci.

[76] Sowers LP, Loo L, Wu Y, Campbell E, Ulrich JD, Wu S, Paemka L, Wassink T, Meyer K, Bing X (2013). Disruption of the non-canonical Wnt gene PRICKLE2 leads to autism-like behaviors with evidence for hippocampal synaptic dysfunction. Mol Psychiatry.

[77] Sperber BR, Boyle-Walsh EA, Engleka MJ, Gadue P, Peterson AC, Stein PL, Scherer SS, McMorris FA (2001). A unique role for Fyn in CNS myelination. J Neurosci.

[78] Spillantini MG, Goedert M (1998). Tau protein pathology in neurodegenerative diseases. Trends Neurosci.

[79] Spillantini MG, Murrell JR, Goedert M, Farlow MR, Klug A, Ghetti B (1998). Mutation in the tau gene in familial multiple system tauopathy with presenile dementia. Proc Natl Acad Sci U S A.

[80] Stuart LM, Bell SA, Stewart CR, Silver JM, Richard J, Goss JL, Tseng AA, Zhang A, El Khoury JB, Moore KJ (2007). CD36 signals to the actin cytoskeleton and regulates microglial migration via a p130Cas complex. J Biol Chem.

[81] Tapia-Rojas C, Cabezas-Opazo F, Deaton CA, Vergara EH, Johnson GVW, Quintanilla RA (2019). It's all about tau. Prog Neurobiol.

[82] Teravskis PJ, Oxnard BR, Miller EC, Kemper L, Ashe KH, Liao D (2019) Phosphorylation in two discrete tau domains regulates a stepwise process leading to postsynaptic dysfunction. J Physiol 10.1113/JP277459 10.1113/JP277459 PMC6908773 31194886

[83] Tezuka T, Umemori H, Akiyama T, Nakanishi S, Yamamoto T (1999). PSD-95 promotes Fyn-mediated tyrosine phosphorylation of the N-methyl-D-aspartate receptor subunit NR2A. Proc Natl Acad Sci U S A.

[84] Um JW, Kaufman AC, Kostylev M, Heiss JK, Stagi M, Takahashi H, Kerrisk ME, Vortmeyer A, Wisniewski T, Koleske AJ (2013). Metabotropic glutamate receptor 5 is a coreceptor for Alzheimer Aβ oligomer bound to cellular prion protein. Neuron.

[85] Um JW, Nygaard HB, Heiss JK, Kostylev MA, Stagi M, Vortmeyer A, Wisniewski T, Gunther EC, Strittmatter SM (2012). Alzheimer amyloid-β oligomer bound to postsynaptic prion protein activates Fyn to impair neurons. Nat Neurosci.

[86] Um JW, Strittmatter SM (2013). Amyloid-β induced signaling by cellular prion protein and Fyn kinase in Alzheimer disease. Prion.

[87] Wemmie JA, Askwith CC, Lamani E, Cassell MD, Freeman JH, Welsh MJ (2003). Acid-sensing ion channel 1 is localized in brain regions with high synaptic density and contributes to fear conditioning. J Neurosci.

[88] Wes PD, Easton A, Corradi J, Barten DM, Devidze N, DeCarr LB, Truong A, He A, Barrezueta NX, Polson C (2014). Tau overexpression impacts a neuroinflammation gene expression network perturbed in Alzheimer's disease. PLoS One.

[89] Williamson R, Scales T, Clark BR, Gibb G, Reynolds CH, Kellie S, Bird IN, Varndell IM, Sheppard PW, Everall I (2002). Rapid tyrosine phosphorylation of neuronal proteins including tau and focal adhesion kinase in response to amyloid-β peptide exposure: involvement of Src family protein kinases. J Neurosci.

[90] Wood JG, Zinsmeister P (1991). Tyrosine phosphorylation systems in Alzheimer's disease pathology. Neurosci Lett.

