# Supplementary material for: Fyn depletion ameliorates tauP301L-induced neuropathology
Source: Acta Neuropathol Commun. 2020 Jul 14;8:108. doi: 10.1186/s40478-020-00979-6 (PMC7362472; doi:10.1186/s40478-020-00979-6)
Supplement: Supplementary file 1 — Additional file 1. Supplementary Information [file 40478_2020_979_MOESM1_ESM.docx]

**Supplementary Information**

| Figure 1 | Fyn KO-AAV (severe hydro) mice had behavioral abnormality  in contextual fear conditioning |
| --- | --- |
| Figure 2 | Fyn KO-AAV mice with severe hydrocephalus had increased  tau hyperphosphorylation |
| Figure 3 | p0 injection of AAV-Tau^P301L^ caused widespread expression of  human tau |
| Figure 4 | Dentate Gyrus had minimal NFT, as detected by Bielschowsky  silver staining |
| Table 1 | Statistical Details for graphs in Fig. 1-7 |


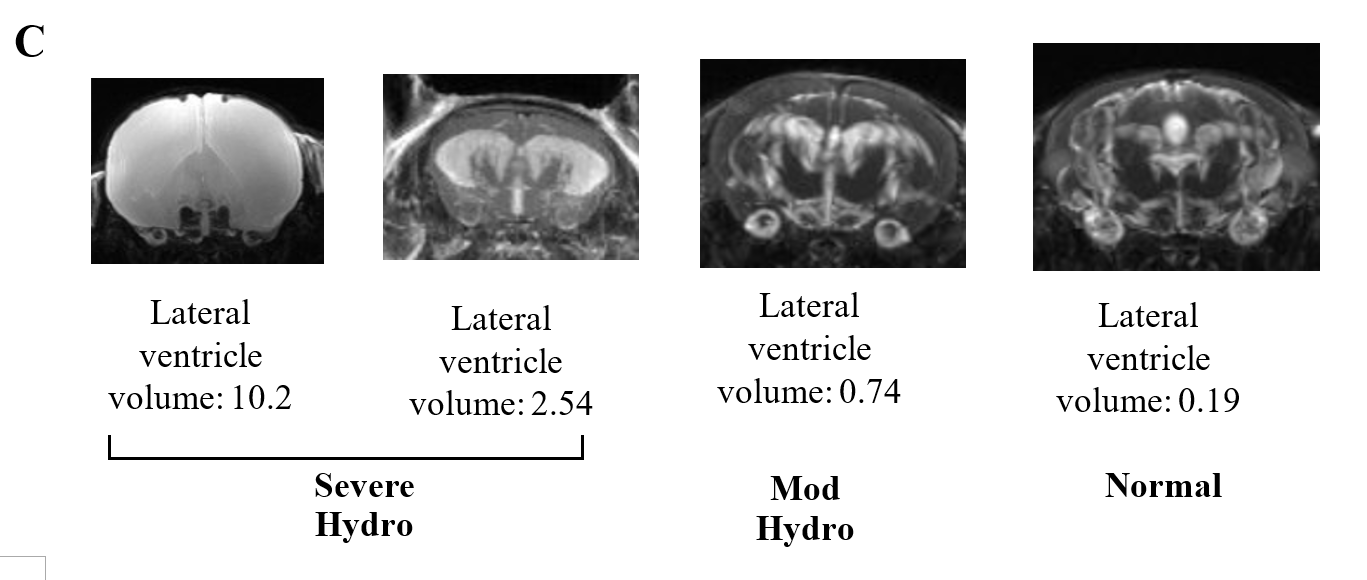


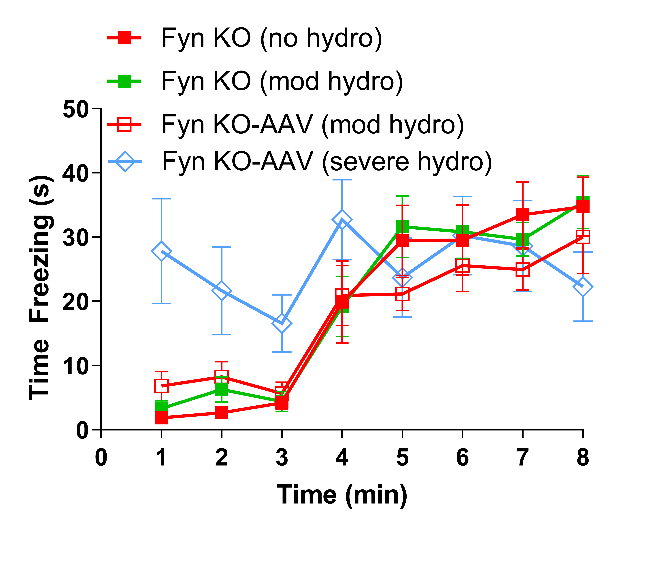

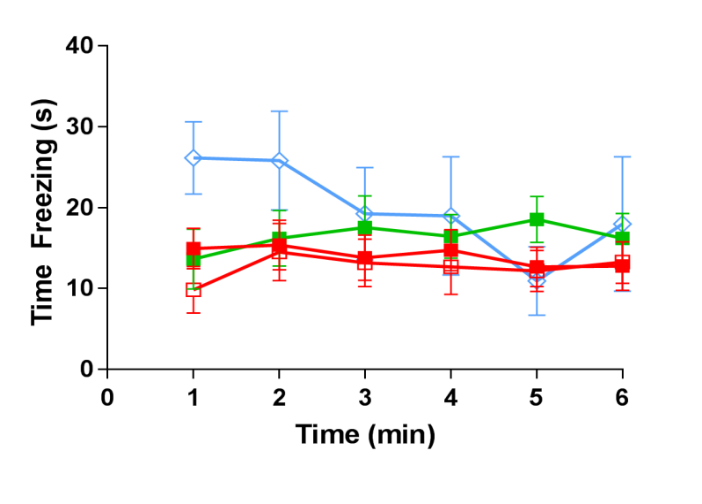


**B**

**C**

***

**A**

*

**SI Figure 1: Fyn KO-AAV (severe hydro) mice had behavior abnormality in contextual fear conditioning**

1. Projections of T2 weighted MRI images of Fyn KO-AAV mice with various levels of hydrocephalus are shown. Note the large amount of CSF (white areas) present in the ventricles in mice with severe hydrocephalus (lateral ventricle volume > 2).
2. Fyn KO-AAV (severe hydro) mice had learning deficit on day 1 of contextual fear conditioning when compared to Fyn KO (no hydro) while there was no difference between Fyn KO-AAV (mod hydro), uninjected Fyn KO (mod hydro) and healthy Fyn KO mice (no hydro) using 2-way ANOVA. ***p=0.0008, *p=0.435. Comparison was made to healthy Fyn KO (no hydro). Fyn KO no hydro n=8, Fyn KO mod hydro n=8: Fyn KO-AAV mod hydro n=9, Fyn KO-AAV severe hydro n=6.
3. No statistically significant differences were detected between the four types of mice on day 2 of contextual fear conditioning.

**B**

**A**

**pS199/pS202/totTau**

**pY18/totTau**

**totTau/GAPDH**

*

**

*

*

**

*

**

**Relative intensity**

**to WT-AAV**

**Relative intensity**

**to WT-AAV**

**Relative intensity**

**to WT-AAV**

Fyn KO-AAV Moderate Hydro

WT-AAV

Fyn KO-AAV Severe Hydro

**SI Figure 2: Fyn KO-AAV mice with severe hydrocephalus had increased Tau hyperphosphorylation**

1. Brain lysates of Fyn KO-AAV with severe hydrocephalus were compared to those of Fyn KO-AAV with moderate hydrocephalus and WT-AAV. Fyn KO-AAV with severe hydrocephalus had dramatically increased pS199/pS202 and pY18 expression (lanes with lysates from severe hydro mice were highlighted with a red box). However, some WT-AAV mice also had dramatically increased pS199/pS202 and pY18 expression despite absence of hydrocephalus.
2. Quantification of total Tau, pS199/S202, and pY18 for WT-AAV and Fyn KO-AAV, with moderate or severe hydrocephalus. Total Tau was normalized to GAPDH, pS199/pS202 to total Tau, and pY18 to total Tau. Black unfilled circle was WT-AAV, red unfilled square Fyn KO-AAV (mod hydro), and blue unfilled diamond Fyn KO-AAV (severe hydro). (Left panel) Relative to WT-AAV, total Tau level was significantly increased in Fyn KO-AAV (severe Hydro) (p=0.0062). Total Tau expression was not significantly different between Fyn KO-AAV (mod hydro) and WT-AAV. (WT-AAV n=22, Fyn KO-AAV (mod hydro) n= 7, Fyn KO-AAV (severe hydro) n= 6). (Center panel) Relative to WT-AAV, pS199/pS202 level was significantly decreased for Fyn KO-AAV (mod hydro) p=0.0375 and increased in Fyn KO-AAV (severe hydro) p=0.0233. Fyn KO-AAV (mod hydro) was also significantly decreased from Fyn KO-AAV (severe hydro) p=0.028. (WT-AAV n=22, Fyn KO-AAV (mod hydro) n= 7, Fyn KO-AAV (severe hydro) n= 7). (Right panel) Relative to WT-AAV, pY18 level was significantly decreased for Fyn KO-AAV (mod hydro) p=0.0155 and increased for Fyn KO-AAV (severe hydro) p=0.0073. Fyn KO-AAV (mod hydro) was also decreased from Fyn KO-AAV (severe hydro) p= 0.0086. (WT-AAV n= 12, Fyn KO-AAV (mod hydro) n= 7, Fyn KO-AAV (severe hydro) n= 7). Unpaired parametric t-test was used.

|  | Total Tau | pS199/S202 | pY18 |
| --- | --- | --- | --- |
| FynKO-AAV (severe) vs WT-AAV | p=0.0062 | p=0.0233 | p=0.0073 |
| FynKO-AAV (mod) vs WT-AAV | p=0.0621 | p=0.0375 | p=0.0155 |
| FynKO-AAV (mod) vs  FynKO-AAV (severe) | p=0.2331 | p=0.028 | p=0.0086 |

**A**

**DD**

**F**

**E**

**BB**

**C**

**SI Figure 3: p0 injection of AAV-Tau^P301L^ caused widespread expression of human Tau**

Low magnification pictures show that AAV-tau^301L^ injection caused widespread human tau expression in the hippocampus of WT and Fyn KO mice (A, B) but not in uninjected WT mice (C). Scale bar 200 µm.

Higher magnification pictures show strong human tau expression in the CA1 and dentate gyrus region in both WT-AAV mice (D) and in the CA3 and dentate gyrus of Fyn KO-AAV mice (E). However, in uninjected WT mice, no human tau was detected (CA1 region, F). Scale bar: 50 µm.


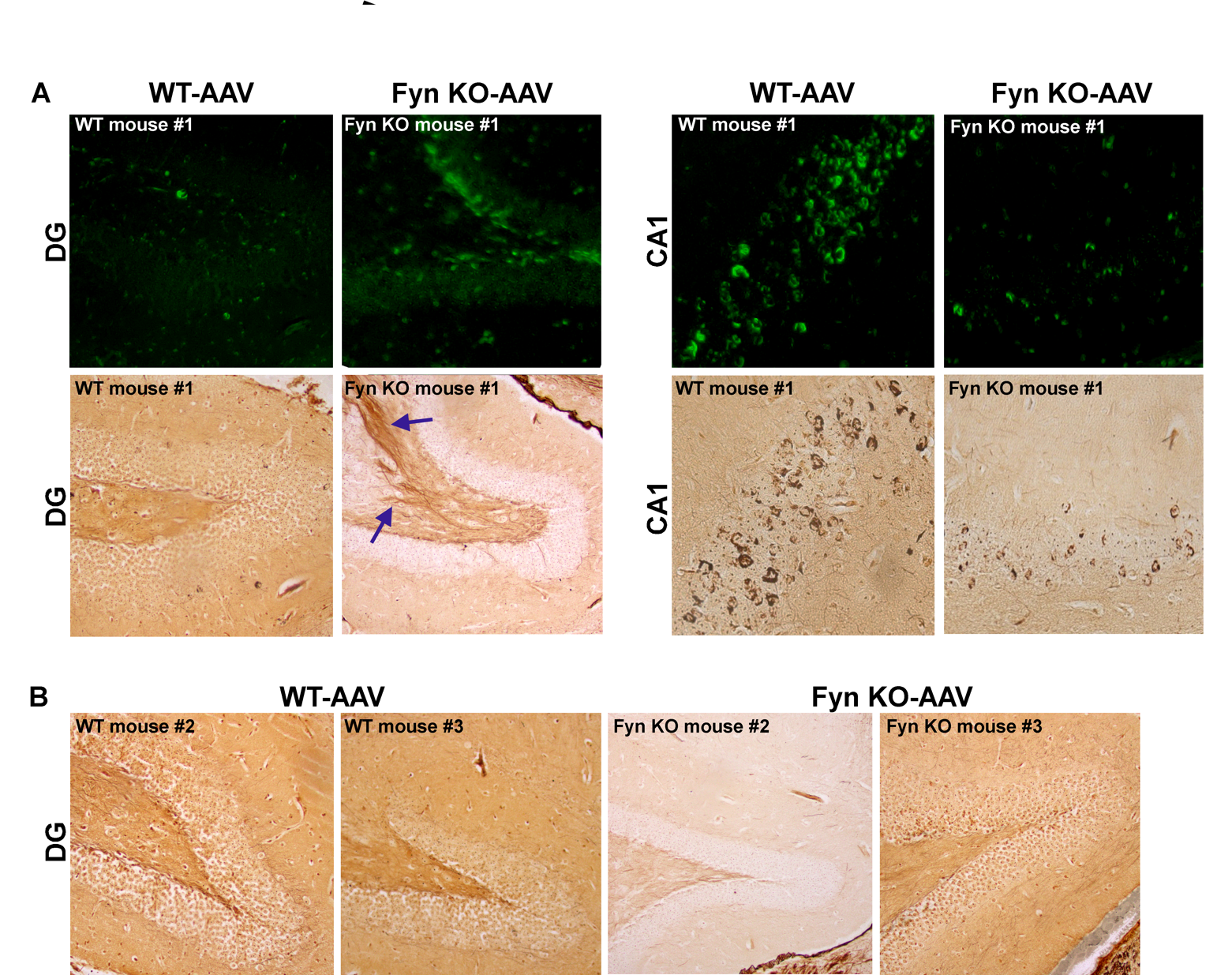


**SI Figure 4: Minimal levels of NFTs in dentate gyrus (DG) of mice expressing tau^P301L^**

1. High magnification pictures show thioflavin S and Bielschowsky silver staining of dentate gyrus in WT-AAV mouse (A, column 1) and Fyn KO-AAV mouse (A, column 2). As a control, similar staining in CA1 is shown in WT-AAV (A, column 3) and Fyn KO-AAV (A, column 4). Adjacent mouse sections were used in thioflavin S and Bielschowsky silver staining.
2. High magnification pictures of the dentate gyrus of two additional WT-AAV and Fyn KO-AAV mice, stained with Bielschowsky silver staining.

Bielschowsky silver stained filaments (arrows) as well as thioflavin S signal were observed in the Fyn KO-AAV mouse #1 but were absent in the Bielschowsky silver stained sections of Fyn KO-AAV mice #2 and #3 as well as WT-AAV mice (SI Fig. 4B).

**Table 1 Statistical Details for graphs in Fig. 1-7**

| **Figure** | **Test** | **t** | **P value** | **DF** |
| --- | --- | --- | --- | --- |
| 1E | t-test | 3.825 | 0.0187 | 4 |
| 2C | t-test | 3.294 | 0.0035 | 21 |
| 2F | t-test | 5.102 | <0.0001 | 22 |
| 2I | t-test | 3.474 | 0.0037 | 14 |
| 3C totTau | non-parametric t-test |  | 0.0532 |  |
| 3C pS199/pS202 | t-test | 2.594 | 0.0106 | 22 |
| 3C pY18 | t-test | 2.652 | 0.0168 | 17 |
| 7C Fyn | t-test | 3.654 | 0.0024 | 15 |

| **Fig 4 C Two-way ANOVA** | | | |
| --- | --- | --- | --- |
| **Source of Variation** | **% of total variation** | **P value** | **P value summary** |
| Interaction | 25.36 | 0.0008 | *** |
| AAV | 35.45 | 0.0003 | *** |
| genotype | 31.85 | 0.0004 | *** |
|  |  |  |  |
| **ANOVA table** | **F (DFn, DFd)** | **P value** | **DF** |
| Interaction | F (1, 8) = 27.64 | P=0.0008 | 1 |
| AAV | F (1, 8) = 38.63 | P=0.0003 | 1 |
| genotype | F (1, 8) = 34.71 | P=0.0004 | 1 |
| Residual |  |  | 8 |

| **Tukey's multiple comparisons test** | **Summary** | **Adjusted P Value** |  |  |
| --- | --- | --- | --- | --- |
| WT vs. Fyn KO | ns | 0.9681 |  |  |
| WT vs. WT-AAV | *** | 0.0002 |  |  |
| WT vs. Fyn KO-AAV | ns | 0.9954 |  |  |
| Fyn KO vs. WT-AAV | *** | 0.0001 |  |  |
| Fyn KO vs.Fyn KO-AAV | ns | 0.9027 |  |  |
| WT-AAV vs. Fyn KO-AAV | *** | 0.0002 |  |  |
|  |  |  |  |  |
| **Fig 4D**  **Two-way ANOVA** | | | | |
| **Source of Variation** | **% of total variation** | **P value** |  | **P value summary** |
| Interaction | 27.4 | 0.0233 |  | * |
| AAV | 44.33 | 0.0074 |  | ** |
| genotype | 0.2551 | 0.7941 |  | ns |
|  |  |  |  |  |
| **ANOVA table** | **F (DFn, DFd)** | **P value** |  | **DF** |
| Interaction | F (1, 8) = 7.823 | P=0.0233 |  | 1 |
| AAV | F (1, 8) = 12.66 | P=0.0074 |  | 1 |
| genotype | F (1, 8) = 0.07284 | P=0.7941 |  | 1 |
| Residual |  |  |  | 8 |
|  |  |  |  |  |
|  |  |  |  |  |
| **Tukey's multiple comparisons test** | **Summary** | **Adjusted P Value** |  |  |
| WT vs. Fyn KO | ns | 0.3452 |  |  |
| WT vs. WT-AAV | ** | 0.0087 |  |  |
| WT vs. Fyn KO-AAV | ns | 0.1712 |  |  |
| Fyn KO vs. WT-AAV | ns | 0.1007 |  |  |
| Fyn KO vs.Fyn KO-AAV | ns | 0.9473 |  |  |
| WT-AAV vs. Fyn KO-AAV | ns | 0.2115 |  |  |

| **Fig 5A Two-way ANOVA** | | | |  |
| --- | --- | --- | --- | --- |
| **Source of Variation** | **% of total variation** | **P value** | **P value summary** |  |
| Interaction | 2.892 | 0.2523 | ns |  |
| aav | 0.09312 | 0.8361 | ns |  |
| genotype | 2.67 | 0.2711 | ns |  |
| **ANOVA table** | **F (DFn, DFd)** | **P value** | **DF** |  |
| Interaction | F (1, 43) = 1.346 | P=0.2523 | 1 |  |
| aav | F (1, 43) = 0.04335 | P=0.8361 | 1 |  |
| genotype | F (1, 43) = 1.243 | P=0.2711 | 1 |  |
| Residual |  |  | 43 |  |
|  |  |  |  |  |
| **Fig 5B Two-way ANOVA** | | | |  |
| **Source of Variation** | **% of total variation** | **P value** | **P value summary** |  |
| Interaction | 7.043 | 0.0367 | * |  |
| AAV | 1.021 | 0.4154 | ns |  |
| genotype | 35.64 | <0.0001 | **** |  |
| **ANOVA table** | **F (DFn, DFd)** | **P value** | **DF** |  |
| Interaction | F (1, 40) = 4.672 | P=0.0367 | 1 |  |
| AAV | F (1, 40) = 0.6772 | P=0.4154 | 1 |  |
| genotype | F (1, 40) = 23.64 | P<0.0001 | 1 |  |
| Residual |  |  | 40 |  |
| **Sidak's multiple comparisons test** | **Summary** | **Adjusted P Value** |  |  |
| WT vs. Fyn KO | *** | 0.0002 |  |  |
| WT vs. WT-AAV | ns | 0.172 |  |  |
| WT vs. Fyn KO-AAV | ** | 0.0049 |  |  |
| Fyn KO vs. WT-AAV | * | 0.0135 |  |  |
| Fyn KO vs.Fyn KO-AAV | ns | 0.94 |  |  |
| WT-AAV vs. Fyn KO-AAV | ns | 0.2634 |  |  |
|  |  |  |  |  |
| **Fig 5B Parametric t-test** | **Test** | **t** | **P value** | **DF** |
| WT vs. WT-AAV | t-test | 2.258 | 0.0333 | 24 |

| **Fig 5C Two-way ANOVA** | | |  |  |
| --- | --- | --- | --- | --- |
| **Source of Variation** | **P value** | **P value summary** |  |  |
| Interaction | 0.5197 | ns |  |  |
| AAV | 0.1857 | ns |  |  |
| genotype | 0.0276 | * |  |  |
| **ANOVA table** | **F (DFn, DFd)** | **P value** | **DF** |  |
| Interaction | F (1, 48) = 0.4207 | P=0.5197 | 1 |  |
| AAV | F (1, 48) = 1.802 | P=0.1857 | 1 |  |
| genotype | F (1, 48) = 5.164 | P=0.0276 | 1 |  |
| Residual |  |  | 48 |  |
| **Tukey's multiple comparisons test** | **Summary** | **Adjusted P Value** |  |  |
| WT vs. Fyn KO | ns | 0.1608 |  |  |
| WT vs. WT-AAV | ns | 0.3364 |  |  |
| WT vs. Fyn KO-AAV | ns | 0.0822 |  |  |
| Fyn KO vs. WT-AAV | ns | 0.9002 |  |  |
| Fyn KO vs.Fyn KO-AAV | ns | 0.9733 |  |  |
| WT-AAV vs. Fyn KO-AAV | ns | 0.6796 |  |  |
|  |  |  |  |  |
| **Fig 5C Parametric t-test** | **Test** | **t** | **P value** | **DF** |
| WT vs.WT-AAV | t-test | 1.887 | 0.0342 | 32 |
| WT vs. Fyn KO | t-test | 3.434 | 0.0022 | 24 |
| WT vs. Fyn KO AAV | t-test | 2.405 | 0.025 | 22 |
| Fyn KO vs Fyn KO AAV | t-test | 0.3645 | 0.7203 | 16 |

| **Fig 5D Two-way ANOVA** | | | |  |
| --- | --- | --- | --- | --- |
| **Source of Variation** | **% of total variation** | **P value** | **P value summary** |  |
| Interaction | 4.336 | 0.0051 | ** |  |
| Row Factor | 46.27 | <0.0001 | **** |  |
| genotype | 3.274 | <0.0001 | **** |  |
| **ANOVA table** | **F (DFn, DFd)** | **P value** | **DF** |  |
| Interaction | F (21, 360) = 2.030 | P=0.0051 | 21 |  |
| Row Factor | F (7, 360) = 64.99 | P<0.0001 | 7 |  |
| genotype | F (3, 360) = 10.73 | P<0.0001 | 3 |  |
| Residual |  |  | 360 |  |
| **Tukey's multiple comparisons test** | **Summary** | **Adjusted P Value** |  |  |
| **Time 1** |  |  |  |  |
| WT vs. WT AAV | ns | 0.9926 |  |  |
| WT vs. Fyn KO | ns | 0.9915 |  |  |
| WT vs. Fyn KO-AAV | ns | 0.7344 |  |  |
| WT AAV vs. Fyn KO | ns | 0.9445 |  |  |
| WT AAV vs. Fyn KO-AAV | ns | 0.5137 |  |  |
| Fyn KO vs. Fyn KO-AAV | ns | 0.9123 |  |  |
| **Time 2** |  |  |  |  |
| WT vs. WT AAV | ns | 0.8907 |  |  |
| WT vs. Fyn KO | ns | 0.9971 |  |  |
| WT vs. Fyn KO-AAV | ns | 0.8189 |  |  |
| WT AAV vs. Fyn KO | ns | 0.8331 |  |  |
| WT AAV vs. Fyn KO-AAV | ns | 0.3741 |  |  |
| Fyn KO vs. Fyn KO-AAV | ns | 0.9311 |  |  |
| **Time 3** |  |  |  |  |
| WT vs. WT AAV | ns | 0.8105 |  |  |
| WT vs. Fyn KO | ns | 0.9498 |  |  |
| WT vs. Fyn KO-AAV | ns | >0.9999 |  |  |
| WT AAV vs. Fyn KO | ns | 0.9976 |  |  |
| WT AAV vs. Fyn KO-AAV | ns | 0.8566 |  |  |
| Fyn KO vs. Fyn KO-AAV | ns | 0.9605 |  |  |
| **Time 4** |  |  |  |  |
| WT vs. WT AAV | ns | 0.1927 |  |  |
| WT vs. Fyn KO | ns | 0.9951 |  |  |
| WT vs. Fyn KO-AAV | ns | >0.9999 |  |  |
| WT AAV vs. Fyn KO | ns | 0.189 |  |  |
| WT AAV vs. Fyn KO-AAV | ns | 0.2962 |  |  |
| Fyn KO vs. Fyn KO-AAV | ns | 0.9929 |  |  |
| **Time 5** |  |  |  |  |
| WT vs. WT AAV | ns | 0.3211 |  |  |
| WT vs. Fyn KO | ns | 0.9994 |  |  |
| WT vs. Fyn KO-AAV | ns | 0.2719 |  |  |
| WT AAV vs. Fyn KO | ns | 0.3691 |  |  |
| WT AAV vs. Fyn KO-AAV | ns | 0.9731 |  |  |
| Fyn KO vs. Fyn KO-AAV | ns | 0.2994 |  |  |
| **Time 6** |  |  |  |  |
| WT vs. WT AAV | ** | 0.0024 |  |  |
| WT vs. Fyn KO | ns | 0.1307 |  |  |
| WT vs. Fyn KO-AAV | ** | 0.009 |  |  |
| WT AAV vs. Fyn KO | ns | 0.8864 |  |  |
| WT AAV vs. Fyn KO-AAV | ns | 0.9948 |  |  |
| Fyn KO vs. Fyn KO-AAV | ns | 0.841 |  |  |
| **Time 7** |  |  |  |  |
| WT vs. WT AAV | ** | 0.0069 |  |  |
| WT vs. Fyn KO | *** | 0.0005 |  |  |
| WT vs. Fyn KO-AAV | *** | 0.0004 |  |  |
| WT AAV vs. Fyn KO | ns | 0.4303 |  |  |
| WT AAV vs. Fyn KO-AAV | ns | 0.4522 |  |  |
| Fyn KO vs. Fyn KO-AAV | ns | 0.9998 |  |  |
| **Time 8** |  |  |  |  |
| WT vs. WT AAV | ** | 0.0052 |  |  |
| WT vs. Fyn KO | ** | 0.0052 |  |  |
| WT vs. Fyn KO-AAV | ** | 0.002 |  |  |
| WT AAV vs. Fyn KO | ns | 0.8845 |  |  |
| WT AAV vs. Fyn KO-AAV | ns | 0.7893 |  |  |
| Fyn KO vs. Fyn KO-AAV | ns | 0.9991 |  |  |

| **Fig 5E Two-way ANOVA** | | |  |
| --- | --- | --- | --- |
| **Source of Variation** | **% of total variation** | **P value** | **P value summary** |
| Time x Genotype | 1.286 | 0.0762 | ns |
| Time | 1.409 | 0.001 | ** |
| Genotype | 43.49 | <0.0001 | **** |
| **AAV** | **40.78** | **<0.0001** | ******** |
| **ANOVA table** | **F (DFn, DFd)** | **P value** | **DF** |
| Time x Genotype | F (15, 230) = 1.595 | P=0.0762 | 15 |
| Time | F (3.469, 159.6) = 5.240 | P=0.0010 | 5 |
| Genotype | F (3, 46) = 16.35 | P<0.0001 | 3 |
| AAV | F (46, 230) = 16.49 | P<0.0001 | 46 |
| Residual |  |  | 230 |
| **Tukey's multiple comparisons test** | **Summary** | **Adjusted P Value** |  |
| **Time 1** |  |  |  |
| WT vs. WT AAV | ns | 0.5953 |  |
| WT vs. Fyn KO | * | 0.0201 |  |
| WT vs. Fyn KO-AAV | ** | 0.0036 |  |
| WT AAV vs. Fyn KO | ns | 0.1344 |  |
| WT AAV vs. Fyn KO-AAV | * | 0.0169 |  |
| Fyn KO vs. Fyn KO-AAV | ns | 0.5534 |  |
| **Time 2** |  |  |  |
| WT vs. WT AAV | ns | 0.0804 |  |
| WT vs. Fyn KO | **** | <0.0001 |  |
| WT vs. Fyn KO-AAV | **** | <0.0001 |  |
| WT AAV vs. Fyn KO | ** | 0.0093 |  |
| WT AAV vs. Fyn KO-AAV | * | 0.01 |  |
| Fyn KO vs. Fyn KO-AAV | ns | 0.9975 |  |
| **Time 3** |  |  |  |
| WT vs. WT AAV | ** | 0.0038 |  |
| WT vs. Fyn KO | **** | <0.0001 |  |
| WT vs. Fyn KO-AAV | **** | <0.0001 |  |
| WT AAV vs. Fyn KO | ** | 0.0034 |  |
| WT AAV vs. Fyn KO-AAV | ** | 0.0027 |  |
| Fyn KO vs. Fyn KO-AAV | ns | 0.9985 |  |
| **Time 4** |  |  |  |
| WT vs. WT AAV | ** | 0.0025 |  |
| WT vs. Fyn KO | **** | <0.0001 |  |
| WT vs. Fyn KO-AAV | **** | <0.0001 |  |
| WT AAV vs. Fyn KO | ** | 0.0047 |  |
| WT AAV vs. Fyn KO-AAV | ** | 0.0045 |  |
| Fyn KO vs. Fyn KO-AAV | ns | 0.9601 |  |
| **Time 5** |  |  |  |
| WT vs. WT AAV | * | 0.0131 |  |
| WT vs. Fyn KO | **** | <0.0001 |  |
| WT vs. Fyn KO-AAV | **** | <0.0001 |  |
| WT AAV vs. Fyn KO | ** | 0.0032 |  |
| WT AAV vs. Fyn KO-AAV | ** | 0.0027 |  |
| Fyn KO vs. Fyn KO-AAV | ns | 0.999 |  |
| **Time 6** |  |  |  |
| WT vs. WT AAV | * | 0.0452 |  |
| WT vs. Fyn KO | **** | <0.0001 |  |
| WT vs. Fyn KO-AAV | **** | <0.0001 |  |
| WT AAV vs. Fyn KO | ** | 0.004 |  |
| WT AAV vs. Fyn KO-AAV | ** | 0.0031 |  |
| Fyn KO vs. Fyn KO-AAV | ns | 0.9993 |  |

| **Fig 6B NeuN Two-way ANOVA** | | | |
| --- | --- | --- | --- |
| **Source of Variation** | **% of total variation** | **P value** | **P value summary** |
| Interaction | 10.28 | 0.0639 | ns |
| Row Factor | 0.457 | 0.6882 | ns |
| Column Factor | 3.448 | 0.2744 | ns |
| **ANOVA table** | **F (DFn, DFd)** | **P value** | **DF** |
| Interaction | F (1, 31) = 3.693 | P=0.0639 | 1 |
| Row Factor | F (1, 31) = 0.1641 | P=0.6882 | 1 |
| Column Factor | F (1, 31) = 1.238 | P=0.2744 | 1 |
| Residual |  |  | 31 |
|  |  |  |  |
| **Fig 6B PSD95 Two-way ANOVA** | | | |
| **Source of Variation** | **% of total variation** | **P value** | **P value summary** |
| Interaction | 17.35 | 0.0043 | ** |
| Row Factor | 8.767 | 0.0357 | * |
| Column Factor | 18.44 | 0.0034 | ** |
| **ANOVA table** | **F (DFn, DFd)** | **P value** | **DF** |
| Interaction | F (1, 29) = 9.604 | P=0.0043 | 1 |
| Row Factor | F (1, 29) = 4.852 | P=0.0357 | 1 |
| Column Factor | F (1, 29) = 10.21 | P=0.0034 | 1 |
| Residual |  |  | 29 |
| **Tukey's multiple comparisons test** | **Summary** | **Adjusted P Value** |  |
| WT vs. Fyn KO | ns | 0.9999 |  |
| WT vs. WT-AAV | ** | 0.0049 |  |
| WT vs. Fyn KO-AAV | ns | 0.9073 |  |
| Fyn KO vs. WT-AAV | ** | 0.0021 |  |
| Fyn KO vs.Fyn KO-AAV | ns | 0.9162 |  |
| WT-AAV vs. Fyn KO-AAV | *** | 0.0005 |  |
|  |  |  |  |
| **Fig 6C NeuN Two-way ANOVA** | | | |
| **Source of Variation** | **% of total variation** | **P value** | **P value summary** |
| Interaction | 10.61 | 0.2996 | ns |
| AAV | 1.5 | 0.6886 | ns |
| genotype | 6.654 | 0.406 | ns |
| **ANOVA table** | **F (DFn, DFd)** | **P value** | **DF** |
| Interaction | F (1, 9) = 1.211 | P=0.2996 | 1 |
| AAV | F (1, 9) = 0.1713 | P=0.6886 | 1 |
| genotype | F (1, 9) = 0.7599 | P=0.4060 | 1 |
| Residual |  |  | 9 |

| **Fig 7B PSD95 Two-way ANOVA** | | | |  |  |
| --- | --- | --- | --- | --- | --- |
| **Source of Variation** | **% of total variation** | **P value** | **P value summary** |  |  |
| Interaction | 0.9902 | 0.476 | ns |  |  |
| Row Factor | 22.49 | 0.0017 | ** |  |  |
| Column Factor | 18.44 | 0.004 | ** |  |  |
| **ANOVA table** | **MS** | **F (DFn, DFd)** | **P value** | **DF** |  |
| Interaction | 0.1188 | F (1, 30) = 0.5210 | P=0.4760 | 1 |  |
| Row Factor | 2.697 | F (1, 30) = 11.83 | P=0.0017 | 1 |  |
| Column Factor | 2.212 | F (1, 30) = 9.702 | P=0.0040 | 1 |  |
| Residual | 0.228 |  |  | 30 |  |
| **Tukey's multiple comparisons test** | **Summary** | **Adjusted P Value** |  |  |  |
| WT vs. Fyn KO | ns | 0.4378 |  |  |  |
| WT vs. WT-AAV | * | 0.027 |  |  |  |
| WT vs. Fyn KO-AAV | ns | 0.9952 |  |  |  |
| Fyn KO vs. WT-AAV | *** | 0.0005 |  |  |  |
| Fyn KO vs.Fyn KO-AAV | ns | 0.2519 |  |  |  |
| WT-AAV vs. Fyn KO-AAV | * | 0.0204 |  |  |  |
|  |  |  |  |  |  |
| **Fig 7B NR2B Two-way ANOVA** | | | |  |  |
| **Source of Variation** | **% of total variation** | **P value** | **P value summary** |  |  |
| Interaction | 9.503 | 0.0554 | ns |  |  |
| Row Factor | 0.2486 | 0.7493 | ns |  |  |
| Column Factor | 12.01 | 0.0326 | * |  |  |
| **ANOVA table** | **F (DFn, DFd)** | **P value** | **DF** |  |  |
| Interaction | F (1, 30) = 3.974 | P=0.0554 | 1 |  |  |
| Row Factor | F (1, 30) = 0.1040 | P=0.7493 | 1 |  |  |
| Column Factor | F (1, 30) = 5.021 | P=0.0326 | 1 |  |  |
| Residual |  |  | 30 |  |  |
| **Tukey's multiple comparisons test** | **Summary** | Adjusted P Value |  |  |  |
| WT vs. Fyn KO | ns | 0.9986 |  |  |  |
| WT vs. WT-AAV | ns | 0.3607 |  |  |  |
| WT vs. Fyn KO-AAV | ns | 0.5071 |  |  |  |
| Fyn KO vs. WT-AAV | ns | 0.314 |  |  |  |
| Fyn KO vs.Fyn KO-AAV | ns | 0.6533 |  |  |  |
| WT-AAV vs. Fyn KO-AAV | ** | 0.0092 |  |  |  |
|  |  |  |  |  |  |
| **Fig 7B NR2B Parametric t-test** | | | | |  |
|  | **Test** | **t** | **P value** | **DF** |  |
| WT vs.WT-AAV | t-test | 2.027 | 0.0304 | 15 |  |
| WT-AAV vs Fyn KO | t-test | 3.208 | 0.0055 | 14 |  |

| **SI Fig 1 B Two-way ANOVA** | | | |
| --- | --- | --- | --- |
| **Source of Variation** | **% of total variation** | **P value** | **P value summary** |
| Interaction | 9.172 | 0.0128 | * |
| Time | 33.77 | <0.0001 | **** |
| hydrocephalus | 3.158 | 0.0041 | ** |
| **ANOVA table** | **F (DFn, DFd)** | **P value** | **DF** |
| Interaction | F (21, 216) = 1.891 | P=0.0128 | 21 |
| Time | F (7, 216) = 20.88 | P<0.0001 | 7 |
| hydrocephalus | F (3, 216) = 4.558 | P=0.0041 | 3 |
| Residual |  |  | 216 |
| **Tukey's multiple comparisons test** | **Summary** | **Adjusted P Value** |  |
| **Time 1** |  |  |  |
| Fyn KO vs. Fyn KO (mod hydro) | ns | 0.9954 |  |
| Fyn KO vs. Fyn KO-AAV (mod hydro) | ns | 0.9224 |  |
| Fyn KO vs. Fyn KO-AAV (severe hydro) | *** | 0.0008 |  |
| Fyn KO (mod hydro) vs. Fyn KO-AAV (mod hydro) | ns | 0.8196 |  |
| Fyn KO (mod hydro) vs. Fyn KO-AAV (severe hydro) | *** | 0.0003 |  |
| Fyn KO-AAV (mod hydro) vs. Fyn KO-AAV (severe hydro) | ** | 0.0043 |  |
| **Time 2** |  |  |  |
| Fyn KO vs. Fyn KO (mod hydro) | ns | 0.9791 |  |
| Fyn KO vs. Fyn KO-AAV (mod hydro) | ns | 0.9391 |  |
| Fyn KO vs. Fyn KO-AAV (severe hydro) | * | 0.0435 |  |
| Fyn KO (mod hydro) vs. Fyn KO-AAV (mod hydro) | ns | 0.7596 |  |
| Fyn KO (mod hydro) vs. Fyn KO-AAV (severe hydro) | * | 0.0155 |  |
| Fyn KO-AAV (mod hydro) vs. Fyn KO-AAV (severe hydro) | ns | 0.1331 |  |
| **Time 3** |  |  |  |
| Fyn KO vs. Fyn KO (mod hydro) | ns | 0.9968 |  |
| Fyn KO vs. Fyn KO-AAV (mod hydro) | ns | 0.9652 |  |
| Fyn KO vs. Fyn KO-AAV (severe hydro) | ns | 0.1387 |  |
| Fyn KO (mod hydro) vs. Fyn KO-AAV (mod hydro) | ns | 0.9939 |  |
| Fyn KO (mod hydro) vs. Fyn KO-AAV (severe hydro) | ns | 0.2045 |  |
| Fyn KO-AAV (mod hydro) vs. Fyn KO-AAV (severe hydro) | ns | 0.288 |  |
| **Time 4** |  |  |  |
| Fyn KO vs. Fyn KO (mod hydro) | ns | 0.9742 |  |
| Fyn KO vs. Fyn KO-AAV (mod hydro) | ns | 0.9938 |  |
| Fyn KO vs. Fyn KO-AAV (severe hydro) | ns | 0.3576 |  |
| Fyn KO (mod hydro) vs. Fyn KO-AAV (mod hydro) | ns | 0.998 |  |
| Fyn KO (mod hydro) vs. Fyn KO-AAV (severe hydro) | ns | 0.1786 |  |
| Fyn KO-AAV (mod hydro) vs. Fyn KO-AAV (severe hydro) | ns | 0.2217 |  |
| **Time 5** |  |  |  |
| Fyn KO vs. Fyn KO (mod hydro) | ns | 0.9973 |  |
| Fyn KO vs. Fyn KO-AAV (mod hydro) | ns | 0.3407 |  |
| Fyn KO vs. Fyn KO-AAV (severe hydro) | ns | 0.6895 |  |
| Fyn KO (mod hydro) vs. Fyn KO-AAV (mod hydro) | ns | 0.457 |  |
| Fyn KO (mod hydro) vs. Fyn KO-AAV (severe hydro) | ns | 0.7947 |  |
| Fyn KO-AAV (mod hydro) vs. Fyn KO-AAV (severe hydro) | ns | 0.976 |  |
| **Time 6** |  |  |  |
| Fyn KO vs. Fyn KO (mod hydro) | ns | 0.9997 |  |
| Fyn KO vs. Fyn KO-AAV (mod hydro) | ns | 0.8581 |  |
| Fyn KO vs. Fyn KO-AAV (severe hydro) | ns | >0.9999 |  |
| Fyn KO (mod hydro) vs. Fyn KO-AAV (mod hydro) | ns | 0.8998 |  |
| Fyn KO (mod hydro) vs. Fyn KO-AAV (severe hydro) | ns | 0.9995 |  |
| Fyn KO-AAV (mod hydro) vs. Fyn KO-AAV (severe hydro) | ns | 0.8738 |  |
| **Time 7** |  |  |  |
| Fyn KO vs. Fyn KO (mod hydro) | ns | 0.4184 |  |
| Fyn KO vs. Fyn KO-AAV (mod hydro) | ns | 0.9998 |  |
| Fyn KO vs. Fyn KO-AAV (severe hydro) | ns | 0.9143 |  |
| Fyn KO (mod hydro) vs. Fyn KO-AAV (mod hydro) | ns | 0.439 |  |
| Fyn KO (mod hydro) vs. Fyn KO-AAV (severe hydro) | ns | 0.8689 |  |
| Fyn KO-AAV (mod hydro) vs. Fyn KO-AAV (severe hydro) | ns | 0.933 |  |
| **Time 8** |  |  |  |
| Fyn KO vs. Fyn KO (mod hydro) | ns | 0.9051 |  |
| Fyn KO vs. Fyn KO-AAV (mod hydro) | ns | 0.9992 |  |
| Fyn KO vs. Fyn KO-AAV (severe hydro) | ns | 0.534 |  |
| Fyn KO (mod hydro) vs. Fyn KO-AAV (mod hydro) | ns | 0.8401 |  |
| Fyn KO (mod hydro) vs. Fyn KO-AAV (severe hydro) | ns | 0.2005 |  |
| Fyn KO-AAV (mod hydro) vs. Fyn KO-AAV (severe hydro) | ns | 0.5901 |  |
|  |  |  |  |
| **SI Fig 1C Two-way ANOVA** | | | |
| **Source of Variation** | **% of total variation** | **P value** | **P value summary** |
| time x Genotype | 5.141 | 0.1818 | ns |
| time | 1.61 | 0.2894 | ns |
| Genotype | 6.509 | 0.3635 | ns |
| time | 52.92 | <0.0001 | **** |
| **ANOVA table** | **F (DFn, DFd)** | **P value** | **DF** |
| time x Genotype | F (15, 135) = 1.349 | P=0.1818 | 15 |
| time | F (3.533, 95.39) = 1.268 | P=0.2894 | 5 |
| Genotype | F (3, 27) = 1.107 | P=0.3635 | 3 |
| time | F (27, 135) = 7.717 | P<0.0001 | 27 |
| Residual |  |  | 135 |
| **Tukey's multiple comparisons test** | **Summary** | **Adjusted P Value** |  |
| **Time 1** |  |  |  |
| Fyn KO vs. Fyn KO mod hydro | ns | 0.9912 |  |
| Fyn KO vs. Fyn KO AAV (mod hydro) | ns | 0.5534 |  |
| Fyn KO vs. Fyn KO AAV (severe hydro) | ns | 0.2071 |  |
| Fyn KO mod hydro vs. Fyn KO AAV (mod hydro) | ns | 0.8479 |  |
| Fyn KO mod hydro vs. Fyn KO AAV (severe hydro) | ns | 0.2 |  |
| Fyn KO AAV (mod hydro) vs. Fyn KO AAV (severe hydro) | ns | 0.0546 |  |
| **Time 2** |  |  |  |
| Fyn KO vs. Fyn KO mod hydro | ns | 0.9978 |  |
| Fyn KO vs. Fyn KO AAV (mod hydro) | ns | 0.9975 |  |
| Fyn KO vs. Fyn KO AAV (severe hydro) | ns | 0.4672 |  |
| Fyn KO mod hydro vs. Fyn KO AAV (mod hydro) | ns | 0.985 |  |
| Fyn KO mod hydro vs. Fyn KO AAV (severe hydro) | ns | 0.548 |  |
| Fyn KO AAV (mod hydro) vs. Fyn KO AAV (severe hydro) | ns | 0.4258 |  |
| **Time 3** |  |  |  |
| Fyn KO vs. Fyn KO mod hydro | ns | 0.864 |  |
| Fyn KO vs. Fyn KO AAV (mod hydro) | ns | 0.9985 |  |
| Fyn KO vs. Fyn KO AAV (severe hydro) | ns | 0.8299 |  |
| Fyn KO mod hydro vs. Fyn KO AAV (mod hydro) | ns | 0.8072 |  |
| Fyn KO mod hydro vs. Fyn KO AAV (severe hydro) | ns | 0.9945 |  |
| Fyn KO AAV (mod hydro) vs. Fyn KO AAV (severe hydro) | ns | 0.7842 |  |
| **Time 4** |  |  |  |
| Fyn KO vs. Fyn KO mod hydro | ns | 0.9676 |  |
| Fyn KO vs. Fyn KO AAV (mod hydro) | ns | 0.9601 |  |
| Fyn KO vs. Fyn KO AAV (severe hydro) | ns | 0.945 |  |
| Fyn KO mod hydro vs. Fyn KO AAV (mod hydro) | ns | 0.8207 |  |
| Fyn KO mod hydro vs. Fyn KO AAV (severe hydro) | ns | 0.9873 |  |
| Fyn KO AAV (mod hydro) vs. Fyn KO AAV (severe hydro) | ns | 0.8609 |  |
| **Time 5** |  |  |  |
| Fyn KO vs. Fyn KO mod hydro | ns | 0.4279 |  |
| Fyn KO vs. Fyn KO AAV (mod hydro) | ns | 0.999 |  |
| Fyn KO vs. Fyn KO AAV (severe hydro) | ns | 0.9841 |  |
| Fyn KO mod hydro vs. Fyn KO AAV (mod hydro) | ns | 0.3749 |  |
| Fyn KO mod hydro vs. Fyn KO AAV (severe hydro) | ns | 0.4827 |  |
| Fyn KO AAV (mod hydro) vs. Fyn KO AAV (severe hydro) | ns | 0.9944 |  |
| **Time 6** |  |  |  |
| Fyn KO vs. Fyn KO mod hydro | ns | 0.8523 |  |
| Fyn KO vs. Fyn KO AAV (mod hydro) | ns | 0.9993 |  |
| Fyn KO vs. Fyn KO AAV (severe hydro) | ns | 0.9323 |  |
| Fyn KO mod hydro vs. Fyn KO AAV (mod hydro) | ns | 0.8845 |  |
| Fyn KO mod hydro vs. Fyn KO AAV (severe hydro) | ns | 0.997 |  |
| Fyn KO AAV (mod hydro) vs. Fyn KO AAV (severe hydro) | ns | 0.9463 |  |
